# An automated plasma protein fractionation design: high-throughput perspectives for proteomic analysis

**DOI:** 10.1186/1756-0500-5-612

**Published:** 2012-11-01

**Authors:** Claudia Boccardi, Silvia Rocchiccioli, Antonella Cecchettini, Alberto Mercatanti, Lorenzo Citti

**Affiliations:** 1 Institute of Clinical Physiology-CNR, Via Moruzzi 1, 56124 Pisa, Italy; 2 Center for Nanotechnology Innovation @NEST, Istituto Italiano di Tecnologia, Piazza San Silvestro 12, 56127 Pisa, Italy; 3 Department of Human Morphology and Applied Biology, University of Pisa, Via Roma 55 Pisa, Italy

**Keywords:** Human plasma, Proteomics, Automated platform, LC-MALDI, High-throughput analysis

## Abstract

**Background:**

Human plasma, representing the most complete record of the individual phenotype, is an appealing sample for proteomics analysis in clinical applications. Up to today, the major obstacle in a proteomics study of plasma is the large dynamic range of protein concentration and the efforts of many researchers focused on the resolution of this important drawback.

**Findings:**

In this study, proteins from pooled plasma samples were fractionated according to their chemical characteristics on a home-designed SPE automated platform. The resulting fractions were digested and further resolved by reversed-phase liquid chromatography coupled with MALDI TOF/TOF mass spectrometry. A total of 712 proteins were successfully identified until a concentration level of ng/mL. Pearson correlation coefficient was used to test reproducibility.

**Conclusions:**

Our multidimensional fractionation approach reduced the analysis time (2 days are enough to process 16 plasma samples filling a 96-well plate) over the conventional gel-electrophoresis or multi-LC column based methods. The robotic processing, avoiding contaminants or lack of sample handling skill, promises highly reproducible specimen analyses (more than 85% Pearson correlation). The automated platform here presented is flexible and easily modulated changing fractioning elements or detectors.

## Finding

### Background

The human blood plasma is the most complex human derived proteome, containing also other tissue proteomes as subsets [1]. Plasma has always been attractive because, besides its easy and safe availability, it represents a complete record of the individual physiological state. In fact, plasma proteome modulations (in terms of concentrations and/or post-translational modifications) reflect biological responses to pathological stimuli and generic homeostasis changes [2]. As a consequence, highly specific biomarkers can be exploited to monitor therapeutic response and their discovery can revolutionize early disease diagnosis and clinical proteomics. On the other hand, plasma/serum is the most difficult protein-containing sample to be characterized mainly due to its dynamic range of concentrations. In addition, clinical proteomics studies are to be systematically conducted and accomplished on large-scale populations in order to obtain a statistical validation. Consequently in this field, sensitivity, reproducibility and sample throughput are necessary and most important conditions in pre-analytical and analytical steps [3,4].

Another big issue arises from the fact that plasma proteins range from serum albumin at the higher abundance level (35–50*10^9^ pg/ml) to interleukin-6 at the lower level (0–5 pg/ml) [1]. In proteomic studies, it is crucial to reduce such an enormous concentration gap by removing or depleting the most represented species, which notoriously mask other proteins. This task is generally faced with immune-affinity techniques that exploit specific antibodies or with cut-off size exclusion methods that ensure a higher reproducibility [5-8].

In regards to this last point, it is also essential to standardize pre-analytical treatment of specimens since blood proteome profile was demonstrated to change according to the nature of anti-coagulant (EDTA, citrate, heparin), centrifugation speed and others [9,10].

Reproducibility together with limited sensitivity are critical limitations also for two dimensional electrophoresis (2DE) which is the widest and most traditional proteome separation method. Though the differential in-gel electrophoresis (DIGE) technique, introduced by Amersham Biosciences-Inc., is aimed at improving reproducibility, the limited applicability of this technology for large-scale samples remains [5,11,12]. More recently, different proteomics strategies, that use multidimensional LC columns coupled with MS/MS analysis, have been developed [13]. These techniques are very sensitive but also expensive and time consuming, especially if applied to high complex mixtures [14-16]. For a better compromise, other emerging methods for protein fractionation employ solid phase extraction (SPE) techniques [17-20]. All these methods are suitable for different mass spectrometry strategies including ESI, SELDI and MALDI [21-23]. Protein profiling using SELDI-TOF-MS has gained over the past few years an increasing interest. SELDI-TOF-MS provides a simple, low-resolution pattern generated from proteins retained on a specific chromatographic surface. Advantages of SELDI are its ability to provide high-throughput and rapid protein expression profiles from complex mixtures with minimal requirements for purification and separation of proteins prior to detection [24]. Nevertheless, SELDI-TOF-MS has some limitations in a routine identification of biomarkers. Additionally, the low resolution, and hence mass accuracy, coupled with the inability to do MS/MS, unlike MALDI-TOF-TOF technology, prevents reliable identification based on conventional bioinformatic searching. Moreover, even if successful at discovering proteins in the low molecular-weight range, it has not yet demonstrated to be consistently successful in studying high molecular weight (HMW) proteins. The SELDI-TOF-MS approach, respect to ESI interface, scrambles to identify hundreds or thousands of proteins in a single analysis [25].

In this paper, a SPE-LC-MS workflow for simultaneous processing of complex protein mixture was presented. This method has been applied on Na-EDTA treated plasma specimens adopting a molecular size cut-off pre-processing [26,27] in order to deplete HMW proteins. Multidimensional liquid chromatography (SPE-type) automated technology has been applied to reduce sample complexity. Following trypsin digestion, peptide samples were fractionated by C18 nano-HPLC chromatography and analysed by MALDI –TOF-TOF mass spectrometry.

The majority of the processing steps have been automatically performed by a robotic station thus preventing sample handling errors.

Selectivity and reproducibility are thoroughly investigated and assessed with mass spectral data.

### Results and discussion

#### Experimental strategy

**Figure 1:**
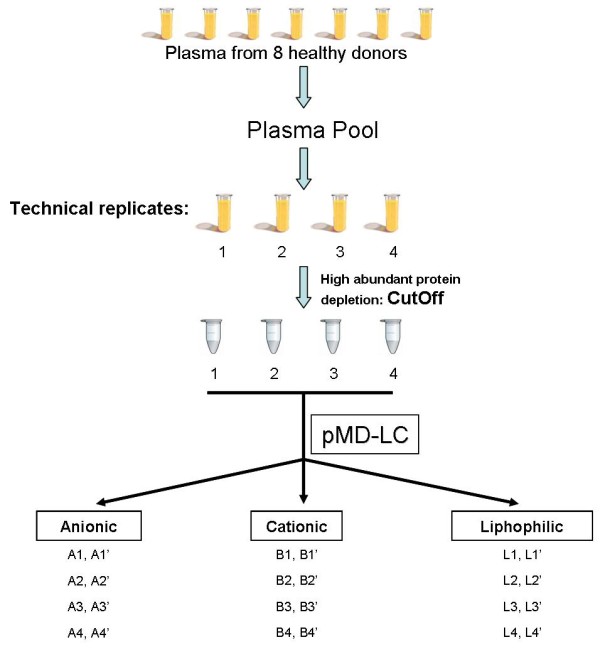
**Workflow overview. **Eight plasma samples from healthy volunteers were collected into a pool to obtain four technical replicates. After cut-off, each replicate was processed by SPE on a robotic device containing three different resins: anionic, cationic and liphophilic. The 24 obtained samples were further fractioned by nanoHPLC-MALDI-TOF-TOF analysis.

Sensitivity, selectivity, reproducibility are the main hurdles in a clinical proteomics study of blood plasma and in order to jump over them a reasoned experimental workflow was designed. Four technical replicates of plasma pooled from healthy volunteers were analysed (Figure 1).

In a first step the most abundant proteins were depleted in order to reduce the dynamic concentration gap. A 30 kDa molecular size membrane cut-off was used to remove HMW proteins [26,27]. This method, although inducing an arbitrary threshold, it is demonstrated to ensure a high reproducibility [28,29], necessary condition for high throughput applications in comparative proteomics analyses (Additional file  1: Figure S1, S2, S3). No significant levels of proteins larger than 50-60 KDa were present in the eluted fraction and reflector mode analysis in the mass range 400-4000 confirmed a significant increase in detection level and an increase of signals which are typically hidden without cut-off. The considerable amount of larger than 60 KDa proteins identified by our method is quite surprising but it could be due to active fragmentation by endogenous proteases in tissues and blood and not to an artefact of the plasma collection or sample preparation. This is a critical aspect that should be taken into consideration in future comparative analyses between physiological or pathological states if proteome and/or degradome profiles of samples need to be compared.

**Figure 2:**
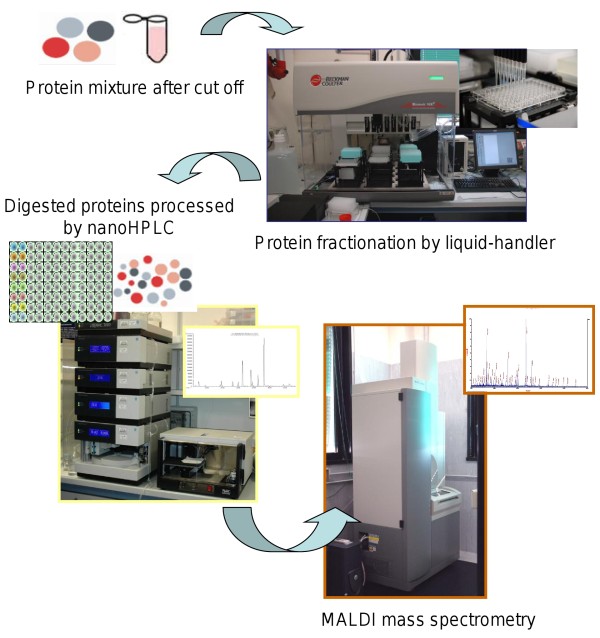
**Steps from cut-off to MALDI analysis. **Photos show the device used to perform the entire sample process.

As a second step of this strategy, we applied and patented a non-conventional automatic, robotic method called parallel Multi Dimensional-Liquid Chromatography (pMD-LC) (patent n° PCT/WO2010/035129 A2). According to this method, the four cut-off selected fractions were simultaneously processed by SPE on a multiple array device containing three distinctive stationary phases (anion and cation phase and lipophilic-phase chromatography) with different binding properties able to trap acid, basic and lipophilic proteins. The automated sample processing was performed with a Liquid Handler Biomek® NX^P^ workstation (Beckman Coulter) and the 96-well plate containing stationary phases was in-house designed and optimized (Additional file  2). Acid and basic fractions were collected on the basis of pH conditions while lipophilic fractions were recovered according to polarity changes of the flushed solution. Protein buffers (mobile phases) were carefully determined and standardized in order to collect fractions in a as small as possible volume. After protein precipitation, all collected protein groups were subjected to a third step to automatically exchange the buffer in order to reduce, alkylate and digest the samples. In the last step, plate containing peptide mixtures was moved to HPLC-autosampler and each sample was loaded on a C18 nano-HPLC column. A good resolution of peaks and a convenient analysis time were obtained using a 30 min H_2_O/TFA/ACN linear elution gradient and each run was collected in 125 final MALDI spots (Figure 2).

Collected spots were subjected to MS and automatic MS/MS analysis to provide accurate protein identification (Additional file  3: Table S1, Additional file  4: Table S2, Additional file  5: Table S3).

#### Comparison with other laboratories

In brief, 5612 peptides matching for more than 596 non redundant proteins were identified (Additional file  3: Table S1, Additional file  4: Table S2, Additional file  5: Table S3). The majority of the proteins were covered by at least 3 peptides (Table 1) as demonstrated by the mean ratio of 3.5 and in fact, only less than 5% of proteins were identified by a single peptide. Table 1 summarizes the obtained results.

**Table 1:** LC-MALDI-TOF-TOF results

**Workflow**	**No. of MS Spectra**^**a**^	**No. of total MS/MS Spectra**	**No. of MS/MS Spectra (mean of all LC-run)**	**Total identified Peptides**	**Non redundant identified peptides**^**b**^	**Mean of proteins ± SD (n total protein)**^**c**^	**MS/MS efficiency (%)**^**d**^	**Peptides/ Proteins ± SD**	**Proteins identified by a single peptide (%)**
Anionic	125	7100	888	2700	1305	163 ± 87.6 (326)	38%	3.8 ± 4.3	15 (4,6%)
Cationic	125	5832	729	1432	718	94 ± 39.6 (188)	26%	3.9 ± 4.4	11 (1,5%)
Lipophilic	125	6554	819	1480	691	99 ± 5.6 (198)	23%	4.0 ± 4.2	9 (1,3%)

a) number of total MS spectra/workflow; b) Peptide counts are based on non- redundant identifications in technical replicates and/or in redundant acquisitions of the same sample; c) Mean ± SD of identified proteins in four and three technical replicates for SPE-LC-MS/MS analysis and in brackets number of total identified proteins; d) Efficiency is determined dividing the number of identified peptides per MS/MS spectra.

Abundant proteins as well as low represented proteins, such as coagulation factor VIII (normal plasma concentration is estimated at about 1 ng/ml), were resolved into 6 order of magnitude of the dynamic concentration range.

As a check of consistency, a comparative analysis of the literature data was accomplished and it evidences that only 30% of proteins in our maps are also identified by Liu and other HUPO laboratories [11,30] (Additional file  1: Table S4, S5).

The low overlapping among different laboratories is due to peculiarities of the plasma samples that is a mixture of different sets of proteins whose assortment depends on individual instantaneous physiological state. The fact that with our approach many identified components appear to be unique demonstrates that different fractionation strategies may disclose distinctive windows of the entire, complex population of proteins as it is also discussed by Liu X et al [30].

#### Selectivity of the methodology

An efficient protein fractionation in sub-families allows a satisfactory protein identification and minimal overlapping.

A total of 596 non-redundant proteins were identified with of only 14 overlapping proteins (2.3%). In particular, the lipophilic fraction shares few proteins with the acid and basic fractions, i.e. 4.5% and 5.7%. A bit higher overlapping of 10% was observed between acid and basic fractions (Additional file  1: Figure S4 and Tables S6–S9).

The low level of protein recurrences among the different sub-sets proves the separation efficiency of solid states and confirms the advantages of the SPE protein fractionation to the classical LC strategies.

The majority of the recovered proteins (172 proteins in anion-exchange and 106 proteins in cation-exchange) displays, as expected, acid and basic pI respectively, this highlighting the chemical specificity of the resins (Additional file  1: Figure S6). pIs are calculated on the native, unmodified proteins while the real pI of the identified species can be affected by post-translational modifications. Additionally the binding charge of the protein to the resin can depend on the chemical characteristic of exposed amino acid residues more than pI. This may explain the incomplete selectivity, based on protein pIs of the ion-exchange resins.

#### Reproducibility within single workflow

In order to perform comparative analyses reproducibility is mandatory.

Sample pools have been used to avoid individual biological variability and thus to assess reproducibility.

UV-profiles reproducibility was checked by a preliminary graphical comparison of retention times and peak intensities on over-layered chromatograms.

For MALDI analysis, shot number and spot coverage are critical parameters to obtain reproducible signal intensity. To overcome the problem of MS signal variations due to heterogeneous sample-matrix co-crystallization, an adequate laser number of shots on each sample spot is necessary, as demonstrated by Hattan *et al*[31]. 5000 total laser shots/spot were chosen. To assess this choice, ACTH 18-39 fragment was used at three different concentrations (5, 10 and 20 fmol/μl), mixed with the matrix and spotted on each spot as internal standard. A linear regression (data not shown) of the signal intensity against peptide concentration (R^2^ = 0,985) evidences a direct correlation between the concentration of the peptide and its relative intensity in the mass spectrum [32]. The concentration of 10 fmol/μl was chosen as a good compromise between the ACTH signal intensity and the need to avoid the signal suppression due to a high concentration of the internal standard.

70% of proteins in the acid-fraction, 65% of proteins obtained in the basic-fraction and 52% of proteins found in the lipophilic fraction are common to all technical replicates (Additional file  3: Table S1, Additional file  4: Table S2, Additional file  5: Table S3) this indicating a good reproducibility of the workflow. The remaining reported identifications to the final number of 596 proteins are detected in at least 3 replicates.

**Figure 3:**
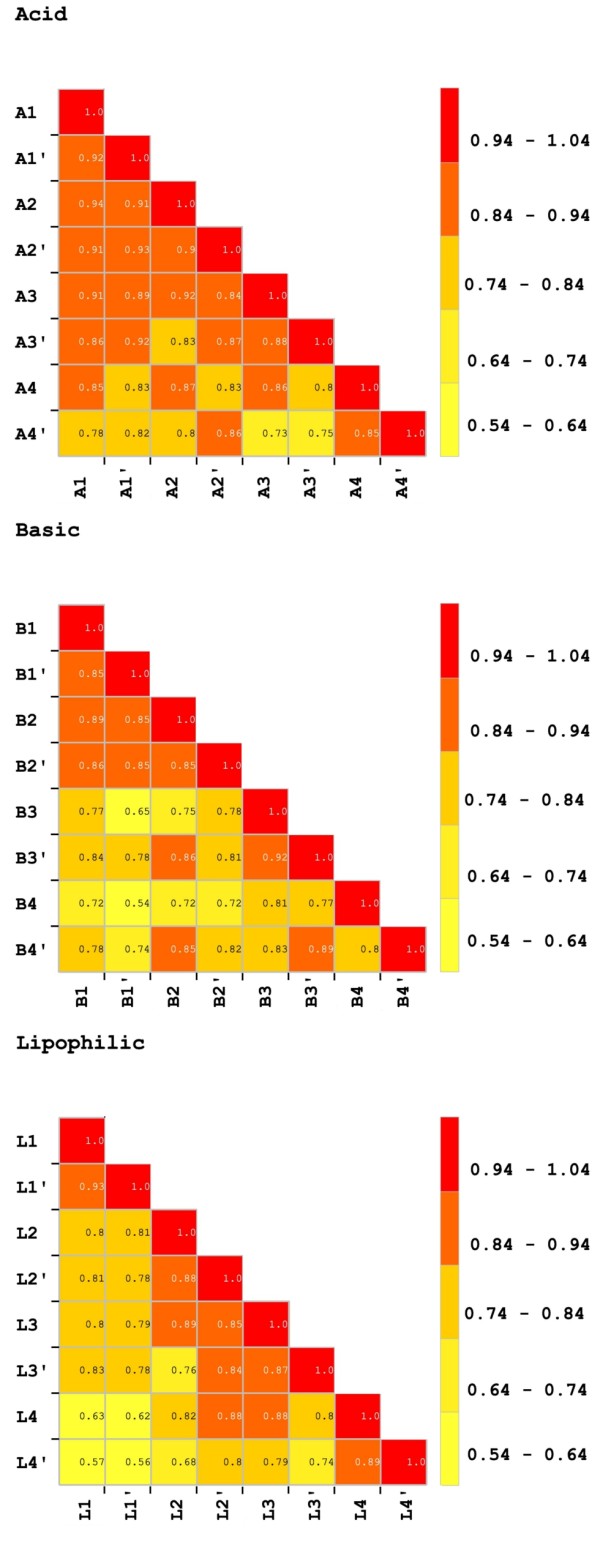
**Similarity matrixes. **Pearson correlation coefficients compare peptide intensities from LC-MALDI-TOF profiles. Peak intensities of each peptide were compared with the same peptide in all other fractions. Four technical replicates for acid, basic, and lipophilic fractions were evaluated.

To analyze the reproducibility within the single method in terms of MS profiles, we compared data obtained by MS features. Reproducibility was also verified in the course of time to assess a potential clinical application of this high-throughput technology. Peak intensities of each peptide (associated by m/z value and retention time in the column) were compared with the same peptide in all other subfamilies creating a matrix of Pearson correlation coefficients to estimate reproducibility (Figure 3).

Data were grouped according to the chemical characteristic of the resin. A good reproducibility of the overall analytical platform is suggested by the good correlation shown in the technical replicates (0,88 ± 0,06 for acid proteins, 0,82 ± 0,09 for basic proteins and 0,82 ± 0,11 for the liphophilic fraction).

The robotic processing of the samples has an important role in improving the reproducibility of the method. Comparing replicates coming from the same cut-off sample (A1 *vs* A1’, A2 *vs* A2’ *etc*) Pearson correlation coefficient is never below 85%. Comparing samples across different cut-offs (A1 *vs* A2 *etc*), a lower correlation is observed. This can be justified considering that cut-off is the only manual step of the workflow.

### Conclusions

The specific aim of this work was to establish an improved method which allows the analysis of a complex mixture of peptides.

The automatic platform and the SPE design, developed to process and analyse complex plasma protein mixtures, results innovative and advantageous and may eventually become a powerful tool for clinical tests in a high throughput screening.

This multidimensional fractionation approach reduces the analysis time (2 days are enough to process 16 plasma samples filling a 96-well plate) and the robotic automation of sample processing, avoiding contaminants, promises high reproducibility (more than 85% Pearson correlation coefficient). Moreover, this automatic platform is flexible and can be freely modulated with different fractioning elements. Since several resins are available, the robotic platform, here described, can be utilized with different design of SPE methods to separate proteins and peptides on the basis of chromatographic interactions.

### Materials and methods

#### Sample collection

Plasma was collected from eight healthy volunteers into Na-EDTA-prepared collection tubes, centrifuged at 750xg for 10 min at room temperature and stored at – 80°C. Identical aliquots of independently pre-processed Na-EDTA treated plasma specimens were pooled and aliquoted in four technical replicates. The study was conducted upon informed consent of volunteers following the approval by the local Ethics University-Hospital Committee in the FIRB-GENOCOR project (Protocol N.48076-study N. 2214).

#### Protein depletion by cut-off

Cut-off was performed with Amicon Ultra-15 (30 KDa; Millipore). The membrane filters were washed with 3 ml of 10% acetonitrile (ACN) and contaminants removed by centrifugation. 1 ml of human plasma pool was diluted with 9 ml of 10% ACN and then loaded on filters. Cut-off units were centrifuged at 5000xg for 90 min at 4°C. 1 ml remaining on the filter was mixed with additional 3 ml of 10% ACN and centrifuged at 5000xg for 90 min. Protein content was quantify by BCA method before and after cut-off. A concentration of 76 mg/ml of plasma proteins was measured in crude plasma samples and this amount was reduced to 1 mg/ml by cut-off depletion. The recovered protein average was 1.3% and represent the ratio between 1 mg of MW < 30KDa proteins and 76 mg of crude plasma. From each sample 12.5 ml of depleted protein solution (about 16 mg) were obtained. Every fraction was splitted in six identical aliquots and lyophilized.

#### Protein fractionation by pMD-LC

Four depleted plasma replicates were independently processed in a device (Liquid handler Beckman Coulter Biomek NX^p^) able to perform an automatic separation using specific reservoirs for protein chemical-trapping (patent n° WO2010/035129 A2). The resins were: BioRad UNOsphere Q Strong Anion Exchange Support, BioRad UNOsphere S Strong Cation Exchange Support and Waters OASIS HLB. Slurries furnished by BioRad and Waters were automatically re-suspended. A constant volume of every resin suspension was transferred into a 96-well filtration plate (Captiva plate-Varian Inc). Two wells were set for each solid state and for each sample. Storage solutions were removed applying vacuum to the filtration plate and recovering the liquid in a 96-well collecting plate. Specific and customized binding solutions (see Additional file  2) were prepared and each stationary phase was washed twice with the same procedure. The 24 lyophilized plasma proteins (6 lyophilized aliquots for each technical replicate) were suspended in 160 μl of binding solution. Each sample was loaded on the top of a stationary single bead packed into the 96-well plate. The binding between resin and proteins was assured alternating vacuum steps with de-pressured steps until the solution was completely adsorbed. After adsorption, each well was washed with binding solution to remove non-specific proteins. Specific and customized eluting solutions (see Additional file  2) were prepared and resins were washed twice eluted fractions which were stored. 600 μl of 7% MeOH/ 7% tButylphosphate/ 86% Acetone were added to the wells and precipitated proteins (100 μg/sample determined by BCA protein concentration assay) from each well were recovered by filtration into Captiva plate-Varian Inc.

#### Reduction, alkylation and digestion

After protein precipitation, all recovered samples were robotically dissolved in 200 μl of 20 mM Ammonium Bicarbonate pH = 8.5 and reduced at 50°C for 20 min by the addition of 10 μl of 0,1 M Dithiothreitol (DTT). 20 μl of 200 mM Iodoacetamide were added and samples incubated at 37°C for 30 min. Trypsin digestion was performed using 10 μl of 0,25 μg/μl enzyme solution (Roche) at 37°C overnight.

#### Reversed phase nano-HPLC

Peptide mixtures from every well were loaded on the auto-sampler module of an Ultimate 3000 nano-HPLC apparatus (Dionex/LC Packings, Sunnyvale, CA, USA) equipped with a 5 mm C18 trapping column (C18 PepMap 100, 5 μm, 100 Å, 300μm id x 5 mm Dionex-LC Packings, Sunnyvale, CA) and with a 77 μm x 150 mm C18 analytical column (3 μm, C18 Dionex-LC Packings, Sunnyvale, CA) equilibrated with 96% of carrier solution A (2% ACN, 0.05% TFA) and 4% eluent solution B (80% ACN, 0.04% TFA). A conventional two steps process was applied: (a) 5 μl of peptide mixture was firstly loaded, concentrated and de-salted in the trapping cartridge flushed with 4% B eluent at flow rate of 20 μl/min and then (b) runned for peptide separation by connection of trap cartridge to capillary analytical column flushed at 300 nl/min under 30 min linear gradient from 4 to 55% of B eluent. Column effluent was monitored by continuous 214 nm absorption recording using a 3-nL UV flow cell. The UV cell out was automatically connected to a robotic device (Probot, Dionex/LC Packings) performing the MALDI matrix addition and eluent spotting directly onto the MALDI plate. The matrix α-CHCA solution (SIGMA) (2 mg/ml in 50% ACN,0.1% TFA), containing also the ACTH peptide fragment (10 fmol/μl ACTH 18-39 clip as an internal mass standard, m/z 2465,199), was added in a 1:2 v/v ratio (eluent to matrix). The collecting of the spots started 12 min after the beginning of LC run. Fractions were directly spotted onto a MALDI plate at 12-s intervals for each spot (60 nl/fraction) at a continuous flow rate of 1.923 μl/min. For each separation run, a total of 125 fractions (spots) were collected.

#### MS and MS/MS processing

MS analyses were performed on a 4800 MALDI-TOF-TOF Analyzer (Applied Biosystems, Foster City, CA, USA), and analytical data were processed and analyzed by a Global Protein Server Workstation (Applied Biosystems), which uses internal Mascot software (Matrix Science, London, UK) to search peptide mass fingerprints and to process MS/MS data. Peak selection was performed in a mass range from 900 to 4000 Da. An exclusion filter was applied to eliminate the internal mass standard and sodium and potassium precursor adducts from the peak list. The top 10 masses in each spot (12-s chromatography time) were then selected for MS/MS analysis. A total of 5000 laser shots were averaged from 50 sample positions. Collision gas was used to generate the high-energy CID spectra using a source voltage of 8 kV, a collision cell voltage of 7 kV, and a second accelerating voltage of 15 kV. Data collection was relatively rapid due to the 200-Hz repetition rate laser and high-speed sample stage. Searches were performed against the IPI protein database for *Homo sapiens*. Database search parameters were: mass tolerance was set at 50 ppm for precursor ion and 0.3 Da for fragment mass value; two missed cleavages were considered for trypsin digestion and variable modifications such as oxidation, de-amidated, and acetyl (N-Terminal) modification were imposed. Only the 2 top-ranked peptide matches were taken into consideration for protein identification. A positive identification was accepted at 97% confidence level or over, meaning that the fragmentation data quality is sufficient to ensure a >97% probability of being assigned to the proper peptide sequence. A confidence interval (CI%) was calculated by GPS Explorer using Mascot ion score and significance, such that a CI% value of 95% or 97%, is equivalent to a Mascot ion score at the significance value. The individual peptide identifications were grouped into protein identifications and assigned a total ion CI% by GPS Explorer [33]. The total ion score CI % is a parameter combining p-value for MS/MS identification with MASCOT search score and considering also the quality of MS/MS analysis by an algorithm developed by AB SCIEX. Proteins were considered as confidentially identified with a significant Mascot score (>40). To eliminate false positive, a search against IPI-random *Homo sapiens* was performed.

#### Data processing

Peak lists were obtained from nanoHPLC-MALDI-TOF chromatograms using a mass tolerance of 0.2 amu, a minimum spectral intensity of 10 and a LC-peak width ≥ 5 sec to select the peaks. For each technical replicate of plasma pool, three peak lists were obtained for acid, basic and lipophilic fractions respectively. All peak list replicates from homogenous protein families were processed by using Marker View version 1.2 software (Applied Biosystems/MDS Sciex, Toronto, Canada) to perform alignments adopting 2 min retention time and 0.2 amu mass tolerance parameters. The obtained mass intensity profiles were imported to Microsoft Excel files and Pearson Correlation Coefficient of every sample was evaluated in comparison with profiles of all the other samples. JColorGrid open source software was then applied for color visualization of the Pearson coefficients.

## Competing interests

The authors declare that there are no competing interests.

## Authors contributions

CB and SR performed sample processing, optimized fractionation conditions and LC-MALDI analyses. AC collected blood samples, prepared plasma, performed cut-off. and carried out manuscript drafting and revising. AM developed and optimized software communications, sample database and data analysis. LC conceived the study. All authors read and approved the final manuscript.

## Supplementary Material

Additional file 1 Supporting figures and tables Additional information about results. Click here for file

Additional file 2 **Supporting materials and methods. **Additional information about solvents, volumes and conditions used for the home-designed robotic platform. Click here for file

Additional file 3 **Supporting Table 1. **Proteins identified in the acid fraction. Protein name, accession number, pI and other additional information are reported. Click here for file

Additional file 4 **Supporting Table 2. **Proteins identified in the basic fraction. Protein name, accession number, pI and other additional information are reported. Click here for file

Additional file 5 **Supporting Table 3. **Proteins identified in the lipophilic fraction. Protein name, accession number, pI and other additional information are reported. Click here for file
