# Supplementary material for: An automated plasma protein fractionation design: high-throughput perspectives for proteomic analysis
Source: BMC Res Notes. 2012 Nov 1;5:612. doi: 10.1186/1756-0500-5-612 (PMC3517536; doi:10.1186/1756-0500-5-612)
Supplement: Additional file 1 — Supporting figures and tables Additional information about results. [file 1756-0500-5-612-S1.doc]

**Supporting Figures and Tables**

**
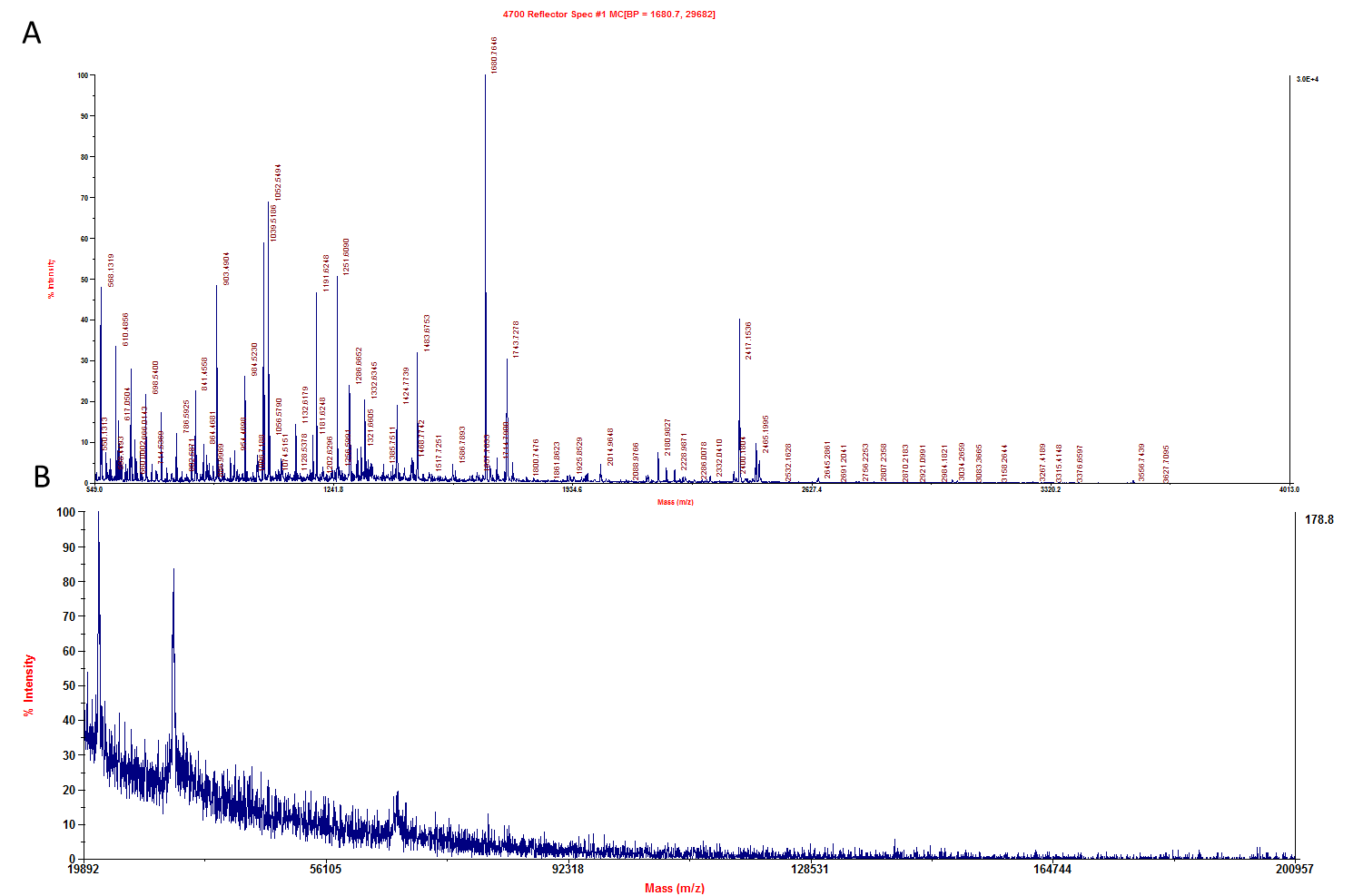
**

**Figure 1:** Plasma sample (pool) after cut off (30KDa): Eluted Fraction.

A) MALDI TOF analysis in positive reflector mode (mass range: 400-4000 Da) B) MALDI TOF analysis in positive linear mode (mass range: 50000-200000)

**
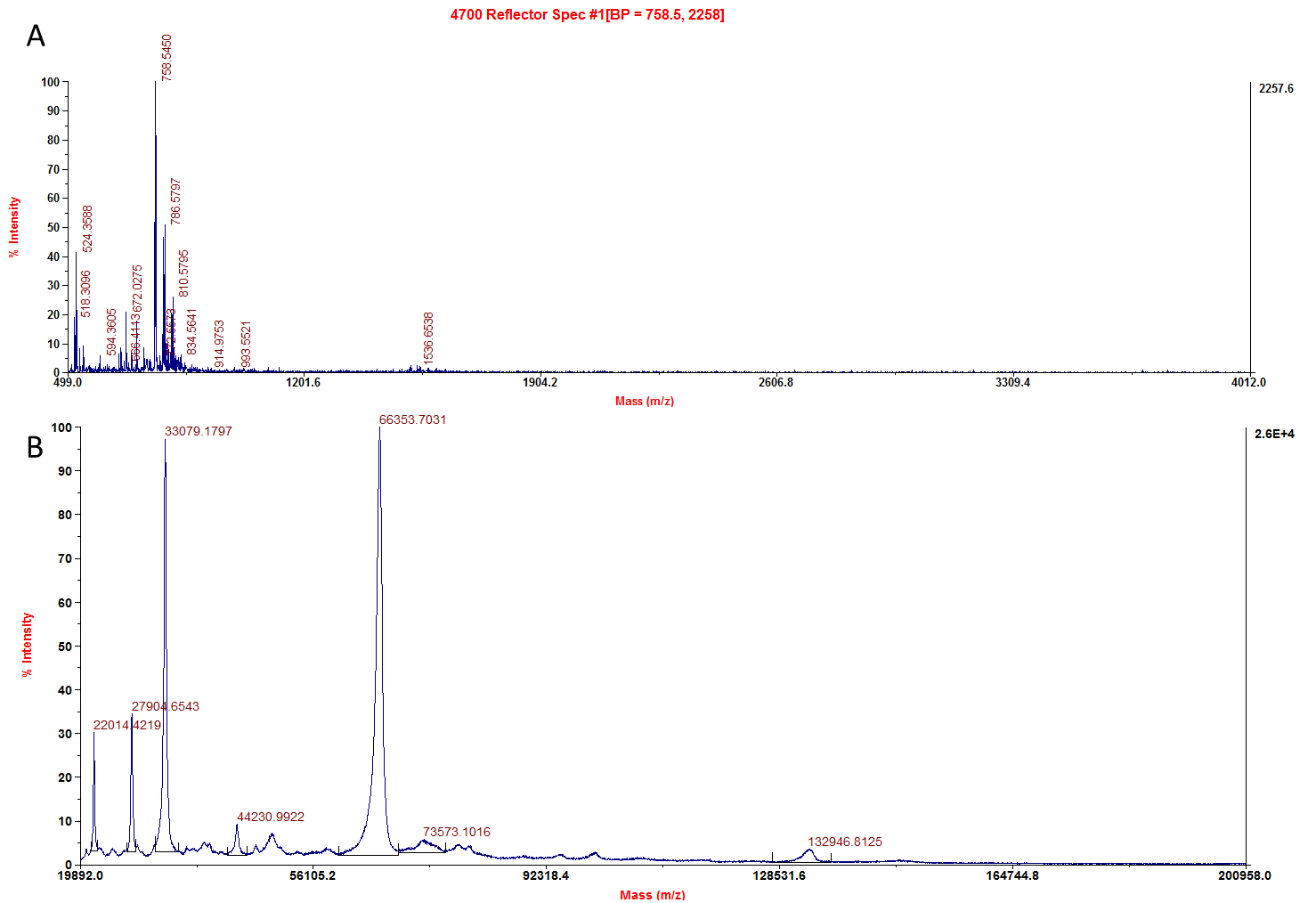
**

**Figure 2:** Plasma samples (pool) after cut off (30KDa): Fraction retained on the filter.

A) MALDI TOF analysis in positive reflector mode (mass range: 400-4000 Da) B) MALDI TOF analysis in positive linear mode (mass range: 50000-200000)


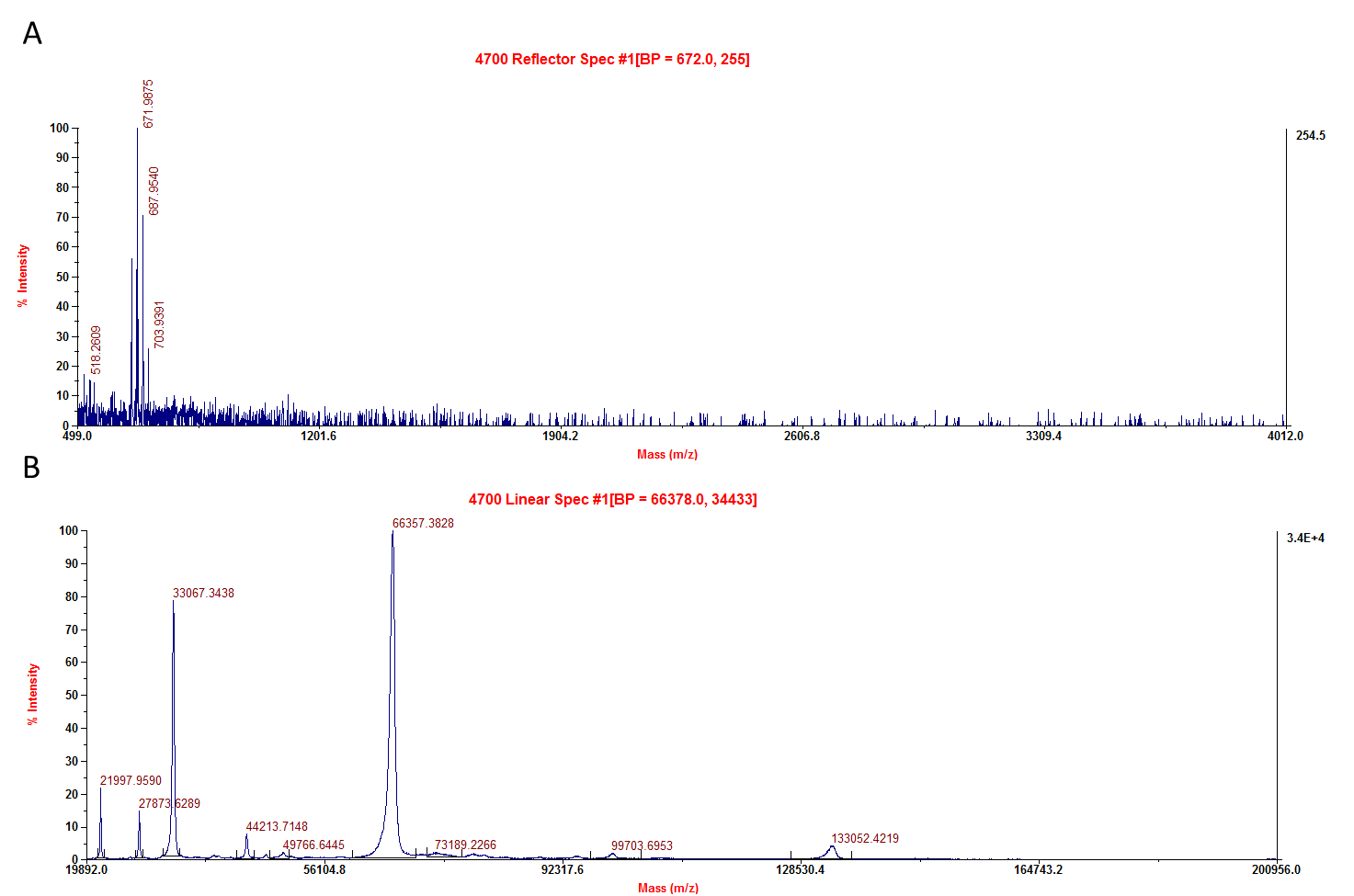


**Figure 3:** Plasma samples (pool) without treatment.

A) MALDI TOF analysis in positive reflecton mode (mass range: 400-4000 Da) B) MALDI TOF analysis in positive linear mode (mass range: 50000-200000)

**Table 4:** Comparison between HUPO PPP identifications (Rai AJ et al: Proteomics 2005, 5, 3262-3277) Liu X et al identifications (Liu X et al: J Am Soc Mass Spectrom 2007, 18, 1249–1264) and our results.

|  | **Total** |
| --- | --- |
| **HUPO PPP identifications ^1^** | **3020** |
| Liu X et al identifications ^2^  (% overlap with HUPOPPP) | 2928  (26%) |
| Boccardi et al identifications  (% overlap with HUPO PPP) | 704  (25%) |

1) HUPO PPP analysis comprised a group of laboratories which applied a 2DE fractionation approach and MALDI TOF analysis for identifications. 2) Liu X. et al applied an SCX-LC-IMS-MS approach.

**Table 5:** Comparison between our results and other labs

|  | **Acid proteins** | % | **Basic proteins** | % | **Lipophilic proteins** | % | **Total** | **%** |
| --- | --- | --- | --- | --- | --- | --- | --- | --- |
| **Boccardi et al identifications** | **326** |  | **189** |  | **197** |  | **704** |  |
| Overlap with HUPO PPP | 74 | **22** | 46 | **24** | 55 | **28** | 175 | 25 |
| Overlap with Liu X et al | 97 | **30** | 56 | **30** | 51 | **26** | 204 | 26 |

**
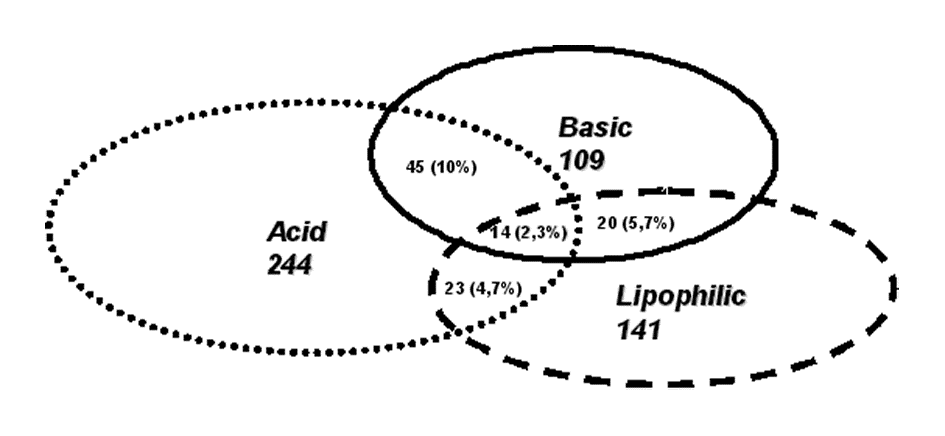
**

**Figure 4:** Venn diagram reporting non- redundant identified proteins in acid, basic and lipophilic fractions.

**Table 6:** Proteins shared between acid- and basic- fractions (45 proteins)

| **Protein Name** | **Accession Number** | **MW** | **PI** |
| --- | --- | --- | --- |
| Gene_Symbol=HERC2 Probable E3 ubiquitin-protein ligase HERC2 | IPI00005826 | 533696,6875 | 5,86 |
| Gene_Symbol=KRT75 cDNA FLJ60809, highly similar to Homo sapiens cytokeratin type II (K6HF) | IPI00005859 | 65720,02344 | 8,52 |
| Gene_Symbol=APC Isoform Long of Adenomatous polyposis coli protein | IPI00012391 | 313619,875 | 7,92 |
| Gene_Symbol=MYH9 Isoform 1 of Myosin-9 | IPI00019502 | 227646,0625 | 5,5 |
| Gene_Symbol=HPX Hemopexin | IPI00022488 | 52384,55078 | 6,55 |
| Gene_Symbol=FAT1 Protocadherin Fat 1 | IPI00031411 | 509684,5 | 4,85 |
| Gene_Symbol=DNM2 Isoform 1 of Dynamin-2 | IPI00033022 | 98345,28906 | 7,04 |
| Gene_Symbol=NPHP4 Nephrocystin-4 | IPI00176920 | 158753,9375 | 8,4 |
| Gene_Symbol=GOLGA4 Isoform 3 of Golgin subfamily A member 4 | IPI00220521 | 261077,6563 | 5,34 |
| Gene_Symbol=RYR1 Isoform 3 of Ryanodine receptor 1 | IPI00334799 | 569891,8125 | 5,18 |
| Gene_Symbol=SSH2 cDNA FLJ58193, highly similar to Protein phosphatase Slingshot homolog 2 | IPI00377071 | 162881,3438 | 5,17 |
| Gene_Symbol=SF1 Isoform 5 of Splicing factor 1 | IPI00386119 | 80796,59375 | 9,55 |
| Gene_Symbol=- Ig kappa chain V-III region B6 | IPI00387113 | 11741,81738 | 9,34 |
| Gene_Symbol=- Ig kappa chain V-III region SIE | IPI00387115 | 11881,88965 | 8,7 |
| Gene_Symbol=IGKC Immunoblobulin light chain (Fragment) | IPI00430808 | 24300,08008 | 8,29 |
| Gene_Symbol=EIF2AK4 Isoform 2 of Eukaryotic translation initiation factor 2-alpha kinase 4 | IPI00456685 | 184898,6875 | 6,03 |
| Gene_Symbol=SLC26A6 Anchor protein | IPI00477896 | 458487,0625 | 6,37 |
| Gene_Symbol=A2M Alpha-2-macroglobulin | IPI00478003 | 164600,3906 | 6 |
| Gene_Symbol=IGHM IGHM protein | IPI00479708 | 69308,70313 | 6,86 |
| Gene_Symbol=POTEE Isoform 1 of ANKRD26-like family C member 1A | IPI00479743 | 122882,2578 | 5,83 |
| Gene_Symbol=SERPINA1 Isoform 1 of Alpha-1-antitrypsin | IPI00553177 | 46878,07813 | 5,37 |
| Gene_Symbol=HPR Isoform 2 of Haptoglobin-related protein | IPI00607707 | 44053,75 | 6,45 |
| Gene_Symbol=NEB Nebulin | IPI00914847 | 775749,4375 | 9,1 |
| Gene_Symbol=BAT2L HLA-B associated transcript 2-like | IPI00741537 | 243957,8125 | 8,55 |
| Gene_Symbol=MYH9 FLJ00279 protein (Fragment) | IPI00742780 | 66014,86719 | 8,97 |
| Gene_Symbol=BAZ2B bromodomain adjacent to zinc finger domain, 2B | IPI00747713 | 242076,4688 | 6,13 |
| Gene_Symbol=C6orf163 Uncharacterized protein C6orf163 | IPI00783612 | 38756,89453 | 6,49 |
| Gene_Symbol=DNAH10 Isoform 1 of Dynein heavy chain 10, axonemal | IPI00784869 | 517677,2188 | 5,64 |
| Gene_Symbol=RGPD8 hypothetical protein | IPI00787969 | 184347,7031 | 6,15 |
| Gene_Symbol=COL7A1 Isoform 2 of Collagen alpha-1(VII) chain | IPI00795118 | 293059,75 | 5,92 |
| Gene_Symbol=RARG 17 kDa protein | IPI00795996 | 18030,96875 | 10,92 |
| Gene_Symbol=LYST Isoform 1 of Lysosomal-trafficking regulator | IPI00796450 | 434169,2813 | 6,15 |
| Gene_Symbol=IGKC IGKC protein | IPI00845354 | 25715,76953 | 6,3 |
| Gene_Symbol=MAP3K1 Mitogen-activated protein kinase kinase kinase 1 | IPI00855985 | 166419,0938 | 7,93 |
| Gene_Symbol=TTLL12 Tubulin tyrosine ligase-like family, member 12 | IPI00879002 | 74184,86719 | 5,34 |
| Gene_Symbol=FRMPD3 similar to FERM and PDZ domain-containing protein 3 | IPI00887257 | 181781,9844 | 8,34 |
| Gene_Symbol=hCG_1774568 similar to hCG1774568 | IPI00887605 | 32051,43555 | 11,81 |
| Gene_Symbol=GUCY2G similar to guanylyl cyclase receptor G | IPI00887656 | 123299,6719 | 9,71 |
| Gene_Symbol=FAT4 Isoform 3 of Protocadherin Fat 4 | IPI00888207 | 546213,9375 | 4,77 |
| Gene_Symbol=- DMXL2 protein | IPI00896496 | 272828,25 | 6,12 |
| Gene_Symbol=HP Haptoglobin | IPI00902590 | 45860,82031 | 6,13 |
| Gene_Symbol=HPR cDNA FLJ31310 fis, clone LIVER1000165, highly similar to Haptoglobin | IPI00902867 | 31672,9707 | 8,48 |
| Gene_Symbol=- cDNA FLJ54284, highly similar to Transcription initiation factor IIF alpha subunit | IPI00909065 | 46409,01953 | 5,75 |
| Gene_Symbol=- cDNA FLJ56758, highly similar to Dolichyl-phosphate beta-glucosyltransferase | IPI00909749 | 25520,36914 | 9,14 |
| Gene_Symbol=- cDNA FLJ59792, highly similar to Homo sapiens outer dense fiber of sperm tails 2-like (ODF2L), transcript variant 1 | IPI00910887 | 68103,32813 | 6,19 |

**Table 7:** Proteins shared between basic- and lipophilic- fractions (20 proteins)

| **Protein Name** | **Accession Number** | **MW** | **PI** |
| --- | --- | --- | --- |
| Gene_Symbol=KRT24 Keratin, type I cytoskeletal 24 | IPI00004550 | 55566,62891 | 4,89 |
| Gene_Symbol=BSN Protein bassoon | IPI00020153 | 418353,9688 | 7,28 |
| Gene_Symbol=KIF13B Kinesin-like protein KIF13B | IPI00021753 | 203908,9531 | 5,56 |
| Gene_Symbol=APC2 Isoform 1 of Adenomatous polyposis coli protein 2 | IPI00025190 | 245965,5938 | 9,08 |
| Gene_Symbol=SPEN Msx2-interacting protein | IPI00045914 | 403030,1875 | 7,35 |
| Gene_Symbol=SSH1 Isoform 3 of Protein phosphatase Slingshot homolog 1 | IPI00103741 | 105974,75 | 5,83 |
| Gene_Symbol=KRT73 Isoform 1 of Keratin, type II cytoskeletal 73 | IPI00174775 | 59456,94922 | 6,93 |
| Gene_Symbol=AKAP9 A-kinase anchor protein 9 | IPI00220624 | 454923,6563 | 4,94 |
| Gene_Symbol=KRT3 Keratin, type II cytoskeletal 3 | IPI00290857 | 64635,66016 | 6,12 |
| Gene_Symbol=NUMA1 Isoform 1 of Nuclear mitotic apparatus protein 1 | IPI00292771 | 239198,625 | 5,63 |
| Gene_Symbol=IRF7 Isoform B of Interferon regulatory factor 7 | IPI00298039 | 52225,10938 | 6,11 |
| Gene_Symbol=CHRD Isoform 1 of Chordin | IPI00306710 | 104703,3516 | 8,07 |
| Gene_Symbol=HMMR hyaluronan-mediated motility receptor isoform a | IPI00337772 | 84575,96875 | 5,73 |
| Gene_Symbol=PRKDC Isoform 2 of DNA-dependent protein kinase catalytic subunit | IPI00376215 | 470161,8125 | 6,81 |
| Gene_Symbol=ZNF844 Zinc finger protein 844 | IPI00397691 | 78707,75781 | 9,14 |
| Gene_Symbol=DAAM2 Disheveled-associated activator of morphogenesis 2 | IPI00514893 | 124275,6797 | 6,36 |
| Gene_Symbol=CEP135 Isoform 1 of Centrosomal protein of 135 kDa | IPI00550987 | 133878,0313 | 5,87 |
| Gene_Symbol=ATR Isoform 2 of Serine/threonine-protein kinase ATR | IPI00554573 | 297450,5 | 7,16 |
| Gene_Symbol=KRT18 Keratin, type I cytoskeletal 18 | IPI00554788 | 48028,53125 | 5,34 |
| Gene_Symbol=- cDNA FLJ58398, highly similar to A-kinase anchor protein 8 | IPI00910261 | 65636,02344 | 4,97 |

**Table 8:** Proteins shared between acid- and lipophilic- fractions (23 proteins)

| **Protein Name** | **Accession Number** | **MW** | **PI** |
| --- | --- | --- | --- |
| Gene_Symbol=LRBA Lipopolysaccharide-responsive and beige-like anchor protein | IPI00002255 | 321637,25 | 5,4 |
| Gene_Symbol=MKI67 Isoform Long of Antigen KI-67 | IPI00004233 | 360697,5313 | 9,49 |
| Gene_Symbol=DSP Isoform DPI of Desmoplakin | IPI00013933 | 334020,625 | 6,44 |
| Gene_Symbol=TNRC6A Isoform 2 of Trinucleotide repeat-containing gene 6A protein | IPI00160265 | 182625,7656 | 6,15 |
| Gene_Symbol=CWC22 Nucampholin homolog | IPI00177381 | 106040,7891 | 6,69 |
| Gene_Symbol=TPM1 Isoform 3 of Tropomyosin alpha-1 chain | IPI00216135 | 32855,75 | 4,72 |
| Gene_Symbol=PTK2B Isoform 2 of Protein tyrosine kinase 2 beta | IPI00216435 | 112366,3125 | 5,69 |
| Gene_Symbol=TOP2B Isoform Beta-1 of DNA topoisomerase 2-beta | IPI00217709 | 183517,5156 | 8,22 |
| Gene_Symbol=TOP2A Isoform 3 of DNA topoisomerase 2-alpha | IPI00218753 | 179398,3281 | 8,92 |
| Gene_Symbol=AKAP9 A-kinase anchor protein 9 | IPI00220624 | 454924,5625 | 4,94 |
| Gene_Symbol=GFAP Isoform 2 of Glial fibrillary acidic protein | IPI00383815 | 50371,80078 | 5,53 |
| Gene_Symbol=C2orf16 Uncharacterized protein C2orf16 | IPI00470912 | 225764,8125 | 10,09 |
| Gene_Symbol=DAAM2 Disheveled-associated activator of morphogenesis 2 | IPI00514893 | 124275,6797 | 6,36 |
| Gene_Symbol=EXO1 Isoform 2 of Exonuclease 1 | IPI00556476 | 90201,46875 | 8,69 |
| Gene_Symbol=CDH3 Isoform 2 of Cadherin-3 | IPI00645614 | 87011,85156 | 4,84 |
| Gene_Symbol=HYDIN2;HYDIN Isoform 1 of Hydrocephalus-inducing protein homolog | IPI00647188 | 580926,5625 | 5,74 |
| Gene_Symbol=CEP290 Isoform 1 of Centrosomal protein of 290 kDa | IPI00784201 | 291049,5938 | 5,75 |
| Gene_Symbol=KRT5 cDNA FLJ54081, highly similar to Keratin, type II cytoskeletal 5 | IPI00796776 | 60316,89844 | 5,95 |
| Gene_Symbol=C9orf117 Isoform 2 of Uncharacterized protein C9orf117 | IPI00847343 | 50122,48828 | 9,16 |
| Gene_Symbol=LBA1 Lupus brain antigen 1 homolog | IPI00847543 | 340340,625 | 6,34 |
| Gene_Symbol=CELSR1 Putative uncharacterized protein CELSR1 | IPI00874160 | 334296,5 | 5,58 |
| Gene_Symbol=DNAH1 Isoform 1 of Dynein heavy chain 1, axonemal | IPI00878816 | 497704,3125 | 5,66 |
| Gene_Symbol=- DMXL2 protein | IPI00896496 | 272828,25 | 6,12 |

**Table 9:** Proteins shared between all fractions (14 proteins)

| **Protein Name** | **Accession Number** | **MW** | **PI** |
| --- | --- | --- | --- |
| Gene_Symbol=CEP164 Isoform 1 of Centrosomal protein of 164 kDa | IPI00007293 | 164727,4375 | 5,27 |
| Gene_Symbol=TF Serotransferrin | IPI00022463 | 79280,46875 | 6,81 |
| Gene_Symbol=KIF14 Kinesin-like protein KIF14 | IPI00299554 | 187742,5 | 8,06 |
| Gene_Symbol=OTOP3 Otopetrin-3 | IPI00332628 | 67222,10938 | 8,96 |
| Gene_Symbol=GRM8 Isoform B of Metabotropic glutamate receptor 8 | IPI00396012 | 103139,5469 | 8,49 |
| Gene_Symbol=RNASE2 zinc finger protein 749 | IPI00397740 | 92701,26563 | 9,01 |
| Gene_Symbol=PLEC1 Isoform 3 of Plectin-1 | IPI00398002 | 519655,0938 | 5,59 |
| Gene_Symbol=DYNC1H1 Cytoplasmic dynein 1 heavy chain 1 | IPI00456969 | 534808,875 | 6,01 |
| Gene_Symbol=ALB Isoform 1 of Serum albumin | IPI00745872 | 71317,24219 | 5,92 |
| Gene_Symbol=KRT72 cDNA FLJ50908, highly similar to Homo sapiens keratin protein K6irs (K6IRS2), mRNA | IPI00793641 | 45127,82813 | 5,11 |
| Gene_Symbol=RHBDF1 cDNA FLJ60400, highly similar to Homo sapiens rhomboid family 1 (Drosophila) (RHBDF1) | IPI00852996 | 64499,46094 | 10,27 |
| Gene_Symbol=LOC100134794 similar to keratin 8 | IPI00887509 | 12466,53027 | 10,16 |
| Gene_Symbol=LOC100129958 similar to hCG1643231 | IPI00888053 | 35447,92188 | 8,21 |
| Gene_Symbol=LOC728498 similar to golgi autoantigen, golgin subfamily a, 8A isoform 1 | IPI00888557 | 68545,02344 | 8,92 |

**
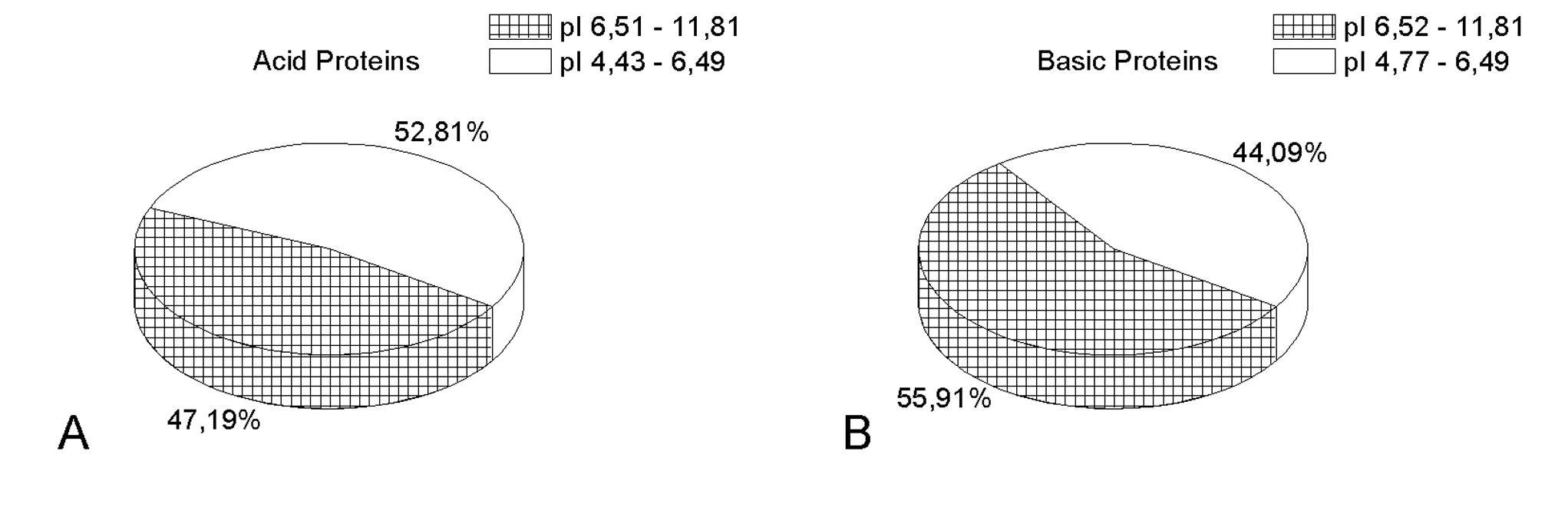
**

**Figure 6:** Distribution of protein pIs for proteins eluted from acid- and basic- fractions
