# Supplementary material for: An automated plasma protein fractionation design: high-throughput perspectives for proteomic analysis
Source: BMC Res Notes. 2012 Nov 1;5:612. doi: 10.1186/1756-0500-5-612 (PMC3517536; doi:10.1186/1756-0500-5-612)
Supplement: Additional file 2 — Supporting materials and methods. Additional information about solvents, volumes and conditions used for the home-designed robotic platform. [file 1756-0500-5-612-S2.doc]

**Supporting Materials and Methods**

160 μl of anion- and cation-exchange resins and 30 μg of Oasis powder resin were deposited in Captiva plate (Varian, Inc. Walnut Creek, CA) and then automatically managed by the liquid handler Biomek NX^P^ (Bekman Coulter, Inc. Brea, CA, USA). The lyophilized plasma samples were suspended with 160 μl of Binding Solution. Table 1 shows the composition of the binding and elution solutions for the three resins.

Each resin was washed twice with the Binding Solution before sample deposition. Prior to protein elution one washing with Binding Solution was made to eliminate non-specific bindings. For the elution: two wash with elution solution were done then combined into one sample. Table 2 shows volumes used to load and detach proteins.

**Table 1: Composition of solutions for the three fractionation resins**

| **Resin** | **Binding Solution** | **Elution Solution** |
| --- | --- | --- |
| Anionic Exchange | 200 mM tris(hydroxymethyl) aminomethane hydrochloride pH=8.5 | 200 mM Formic Acid, Ammonium hydroxide  pH= 2.5 |
| Cationic Exchange | 200 mM Formic acid/NH_3_ pH=5.5 | 5% Ammonium hydroxide, 1M sodium chloride pH=11 |
| Oasis | 5% MeOH | MeOH |

**Table 2: SPE volumes**

|  | **Solution** | **Vol μl** |
| --- | --- | --- |
| **Conditioning** | Binding Sol. | 390 |
|  | Binding Sol. | 650 |
| **Sample** | *Plasma in Binding Sol.* | 160 |
| **Washing** | Binding Sol. | 650 |
| **Extraction** | Elution Sol. | 130 |
|  | Elution Sol. | 130 |

**pMDLC**

**Acid Basic Lipo.**

**A1-4**

**A1’-4’**

**B1-4**

**B1’-4’**

**L1-4**

**L1’-4’**

**1**

**2**

**3**

**4**

**5**

**6**

**7**

**8**

**Sample**

Up to eight samples can be processed simultaneously in the 96-well plate. Acid, basic and lipophilic resins can be loaded into the filtration plate. Many plate configurations can be set.
