# Supplementary material for: An automated plasma protein fractionation design: high-throughput perspectives for proteomic analysis
Source: BMC Res Notes. 2012 Nov 1;5:612. doi: 10.1186/1756-0500-5-612 (PMC3517536; doi:10.1186/1756-0500-5-612)
Supplement: Additional file 3 — Supporting Table 1. Proteins identified in the acid fraction. Protein name, accession number, pI and other additional information are reported. [file 1756-0500-5-612-S3.doc]

| **N** | **Protein Name** | **Acc Number** | **MW** | **PI** | **Peptide Count** | **Total Ion Score** | **Total Ion Score C.I. %** | **N tech repl** |
| --- | --- | --- | --- | --- | --- | --- | --- | --- |
| 1 | Gene_Symbol=PCM1 Isoform 3 of Pericentriolar material 1 protein | IPI00001654 | 234123 | 4,91 | 4 | 46,3 | 99 | 4 |
| 2 | Gene_Symbol=LRBA Lipopolysaccharide-responsive and beige-like anchor protein | IPI00002255 | 321637 | 5,4 | 4 | 48,0 | 99 | 4 |
| 3 | Gene_Symbol=PIK3C2A Phosphatidylinositol-4-phosphate 3-kinase C2 domain-containing alpha polypeptide | IPI00002580 | 192156 | 8,25 | 5 | 64,0 | 100 | 4 |
| 4 | Gene_Symbol=MAPK6 Mitogen-activated protein kinase 6 | IPI00003431 | 83256 | 4,91 | 3 | 47,6 | 99 | 4 |
| 5 | Gene_Symbol=MKI67 Isoform Long of Antigen KI-67 | IPI00004233 | 360698 | 9,49 | 5 | 80,2 | 100 | 4 |
| 6 | Gene_Symbol=HERC2 Probable E3 ubiquitin-protein ligase HERC2 | IPI00005826 | 533697 | 5,86 | 5 | 76,2 | 100 | 4 |
| 7 | Gene_Symbol=KRT75 cDNA FLJ60809, highly similar to Homo sapiens cytokeratin type II (K6HF) | IPI00005859 | 65720 | 8,52 | 6 | 226,2 | 100 | 4 |
| 8 | Gene_Symbol=CEP164 Isoform 1 of Centrosomal protein of 164 kDa | IPI00007293 | 164727 | 5,27 | 3 | 72,0 | 100 | 4 |
| 9 | Gene_Symbol=PADI4 Protein-arginine deiminase type-4 | IPI00008307 | 75131 | 6,15 | 3 | 48,6 | 100 | 3 |
| 10 | Gene_Symbol=KRT76 Keratin, type II cytoskeletal 2 oral | IPI00008359 | 66400 | 8,38 | 4 | 128,9 | 100 | 3 |
| 11 | Gene_Symbol=RCOR1 REST corepressor 1 | IPI00008531 | 53110 | 6,56 | 1 | 47,4 | 99 | 3 |
| 12 | Gene_Symbol=NRL Neural retina-specific leucine zipper protein | IPI00008875 | 26095 | 7,74 | 2 | 44,0 | 99 | 4 |
| 13 | Gene_Symbol=EFCAB6 Isoform 1 of EF-hand calcium-binding domain-containing protein 6 | IPI00009724 | 174702 | 8,67 | 6 | 65,3 | 100 | 4 |
| 14 | Gene_Symbol=TLR7 Toll-like receptor 7 | IPI00009812 | 122384 | 8,51 | 4 | 86,5 | 100 | 3 |
| 15 | Gene_Symbol=KRT10 Keratin, type I cytoskeletal 10 | IPI00009865 | 59703 | 5,13 | 17 | 872,6 | 100 | 4 |
| 16 | Gene_Symbol=MXRA5 Matrix-remodeling-associated protein 5 | IPI00012347 | 314195 | 8,57 | 3 | 43,9 | 99 | 3 |
| 17 | Gene_Symbol=APC Isoform Long of Adenomatous polyposis coli protein | IPI00012391 | 313620 | 7,92 | 4 | 54,9 | 100 | 4 |
| 18 | Gene_Symbol=CPT2 Carnitine O-palmitoyltransferase 2, mitochondrial | IPI00012912 | 74244 | 8,38 | 3 | 60,2 | 100 | 3 |
| 19 | Gene_Symbol=BIRC2 Baculoviral IAP repeat-containing protein 2 | IPI00013418 | 71109 | 6,27 | 2 | 40,6 | 97 | 4 |
| 20 | Gene_Symbol=DSP Isoform DPI of Desmoplakin | IPI00013933 | 334021 | 6,44 | 6 | 58,5 | 100 | 4 |
| 21 | Gene_Symbol=ZFHX3 Isoform A of Zinc finger homeobox protein 3 | IPI00014186 | 408841 | 5,82 | 3 | 46,8 | 99 | 4 |
| 22 | Gene_Symbol=KRT12 Keratin, type I cytoskeletal 12 | IPI00015309 | 53592 | 4,7 | 3 | 88,6 | 100 | 4 |
| 23 | Gene_Symbol=F8 Coagulation factor VIII | IPI00017603 | 268323 | 6,95 | 4 | 51,7 | 100 | 4 |
| 24 | Gene_Symbol=IFT74 Intraflagellar transport protein 74 homolog | IPI00018090 | 69310 | 5,73 | 3 | 41,3 | 98 | 4 |
| 25 | Gene_Symbol=GLI3 Zinc finger protein GLI3 | IPI00018889 | 171396 | 7,02 | 3 | 56,6 | 100 | 4 |
| 26 | Gene_Symbol=KRT9 Keratin, type I cytoskeletal 9 | IPI00019359 | 62320 | 5,19 | 11 | 430,1 | 100 | 4 |
| 27 | Gene_Symbol=MYH9 Isoform 1 of Myosin-9 | IPI00019502 | 227646 | 5,5 | 6 | 83,7 | 100 | 4 |
| 28 | Gene_Symbol=ASS1 Argininosuccinate synthase | IPI00020632 | 51184 | 8,6 | 2 | 56,8 | 100 | 4 |
| 29 | Gene_Symbol=AHNAK Neuroblast differentiation-associated protein AHNAK | IPI00021812 | 629218 | 5,8 | 6 | 78,4 | 100 | 4 |
| 30 | Gene_Symbol=APOA1 Apolipoprotein A-I | IPI00021841 | 30759 | 5,56 | 5 | 172,9 | 100 | 4 |
| 31 | Gene_Symbol=HRG Histidine-rich glycoprotein | IPI00022371 | 60510 | 7,09 | 5 | 120,4 | 100 | 3 |
| 32 | Gene_Symbol=AFP Alpha-fetoprotein | IPI00022443 | 70458 | 5,48 | 1 | 62,4 | 100 | 4 |
| 33 | Gene_Symbol=DOCK2 Isoform 1 of Dedicator of cytokinesis protein 2 | IPI00022449 | 213123 | 6,43 | 4 | 40,5 | 97 | 4 |
| 34 | Gene_Symbol=TF Serotransferrin | IPI00022463 | 79280 | 6,81 | 41 | 3016,3 | 100 | 4 |
| 35 | Gene_Symbol=HERC1 Probable E3 ubiquitin-protein ligase HERC1 | IPI00022479 | 538918 | 5,69 | 2 | 42,8 | 98 | 4 |
| 36 | Gene_Symbol=HPX Hemopexin | IPI00022488 | 52385 | 6,55 | 6 | 244,8 | 100 | 4 |
| 37 | Gene_Symbol=A1BG Alpha-1B-glycoprotein | IPI00022895 | 54809 | 5,58 | 3 | 95,4 | 100 | 4 |
| 38 | Gene_Symbol=LRP2 Low-density lipoprotein receptor-related protein 2 | IPI00024292 | 540376 | 4,89 | 5 | 52,1 | 100 | 4 |
| 39 | Gene_Symbol=KIF15 Isoform 1 of Kinesin-like protein KIF15 | IPI00024975 | 161030 | 5,75 | 3 | 62,1 | 100 | 4 |
| 40 | Gene_Symbol=FLG Filaggrin | IPI00026256 | 435036 | 9,24 | 4 | 55,6 | 100 | 3 |
| 41 | Gene_Symbol=SEC23IP Isoform 1 of SEC23-interacting protein | IPI00026969 | 111691 | 5,35 | 3 | 44,9 | 99 | 3 |
| 42 | Gene_Symbol=GALNT6 Polypeptide N-acetylgalactosaminyltransferase 6 | IPI00026991 | 72025 | 8,47 | 2 | 42,1 | 98 | 3 |
| 43 | Gene_Symbol=GRIN2A Glutamate [NMDA] receptor subunit epsilon-1 | IPI00029768 | 166489 | 6,67 | 4 | 57,0 | 100 | 4 |
| 44 | Gene_Symbol=GOLGA1 Golgin subfamily A member 1 | IPI00031115 | 88260 | 5,24 | 3 | 43,6 | 99 | 4 |
| 45 | Gene_Symbol=FRAP1 FKBP12-rapamycin complex-associated protein | IPI00031410 | 290759 | 6,73 | 4 | 46,2 | 99 | 3 |
| 46 | Gene_Symbol=FAT1 Protocadherin Fat 1 | IPI00031411 | 509685 | 4,85 | 2 | 45,5 | 99 | 4 |
| 47 | Gene_Symbol=DNM2 Isoform 1 of Dynamin-2 | IPI00033022 | 98345 | 7,04 | 2 | 50,7 | 100 | 4 |
| 48 | Gene_Symbol=PTPN23 Tyrosine-protein phosphatase non-receptor type 23 | IPI00034006 | 179830 | 6,45 | 3 | 41,1 | 97 | 4 |
| 49 | Gene_Symbol=CEP63 Isoform 4 of Centrosomal protein of 63 kDa | IPI00041779 | 55841 | 6,1 | 4 | 86,6 | 100 | 4 |
| 50 | Gene_Symbol=ZNF407 Isoform 1 of Zinc finger protein 407 | IPI00043516 | 251658 | 6,05 | 6 | 68,4 | 100 | 4 |
| 51 | Gene_Symbol=KRT71 Keratin, type II cytoskeletal 71 | IPI00061200 | 57727 | 6,28 | 3 | 61,6 | 100 | 4 |
| 52 | Gene_Symbol=FRMPD3 FERM and PDZ domain-containing protein 3 | IPI00064201 | 200852 | 8,44 | 3 | 43,9 | 99 | 3 |
| 53 | Gene_Symbol=CCDC27 Coiled-coil domain-containing protein 27 | IPI00065310 | 75821 | 5,53 | 2 | 40,5 | 97 | 4 |
| 54 | Gene_Symbol=CCDC11 Coiled-coil domain-containing protein 11 | IPI00065428 | 62195 | 9 | 4 | 47,5 | 99 | 3 |
| 55 | Gene_Symbol=C9orf43 cDNA FLJ56263 | IPI00152071 | 54834 | 9,01 | 3 | 60,7 | 100 | 3 |
| 56 | Gene_Symbol=DNAH3 Isoform 1 of Dynein heavy chain 3, axonemal | IPI00152462 | 473776 | 6,04 | 6 | 95,3 | 100 | 4 |
| 57 | Gene_Symbol=DNAH5 Dynein heavy chain 5, axonemal | IPI00152653 | 532504 | 5,79 | 4 | 46,0 | 99 | 4 |
| 58 | Gene_Symbol=TNRC6A Isoform 2 of Trinucleotide repeat-containing gene 6A protein | IPI00160265 | 182626 | 6,15 | 3 | 41,2 | 98 | 4 |
| 59 | Gene_Symbol=IFT81 Isoform CDV-1R of Intraflagellar transport protein 81 homolog | IPI00165189 | 80038 | 8,9 | 2 | 44,0 | 99 | 3 |
| 60 | Gene_Symbol=CHD9 14 kDa protein | IPI00166739 | 14013 | 9,65 | 2 | 41,4 | 98 | 3 |
| 61 | Gene_Symbol=NARG1L Isoform 1 of NMDA receptor-regulated 1-like protein | IPI00167368 | 102424 | 8,06 | 3 | 41,2 | 98 | 3 |
| 62 | Gene_Symbol=FHAD1 Isoform 3 of Forkhead-associated domain-containing protein 1 | IPI00167447 | 167280 | 6,82 | 4 | 51,1 | 100 | 3 |
| 63 | Gene_Symbol=WDR36 WD repeat-containing protein 36 | IPI00169325 | 106282 | 7,32 | 4 | 46,0 | 99 | 3 |
| 64 | Gene_Symbol=NPHP4 Nephrocystin-4 | IPI00176920 | 158754 | 8,4 | 3 | 73,3 | 100 | 4 |
| 65 | Gene_Symbol=CWC22 Nucampholin homolog | IPI00177381 | 106041 | 6,69 | 3 | 50,5 | 100 | 4 |
| 66 | Gene_Symbol=ALMS1 | IPI00178743 | 463304 | 5,86 | 4 | 76,1 | 100 | 4 |
| 67 | Gene_Symbol=ROBO4 Putative uncharacterized protein ROBO4 | IPI00179778 | 86207 | 9,06 | 2 | 49,6 | 100 | 3 |
| 68 | Gene_Symbol=MYH15 Myosin-15 | IPI00180408 | 225929 | 5,66 | 4 | 52,9 | 100 | 4 |
| 69 | Gene_Symbol=MYO3A Myosin IIIA | IPI00185036 | 187574 | 9,04 | 4 | 48,6 | 100 | 3 |
| 70 | Gene_Symbol=PTK2B Isoform 2 of Protein tyrosine kinase 2 beta | IPI00216435 | 112366 | 5,69 | 4 | 40,5 | 97 | 4 |
| 71 | Gene_Symbol=SPTBN4 Isoform 4 of Spectrin beta chain, brain 3 | IPI00217048 | 244471 | 5,47 | 4 | 42,4 | 98 | 4 |
| 72 | Gene_Symbol=KCNK10 Isoform C of Potassium channel subfamily K member 10 | IPI00217521 | 60640 | 9,12 | 2 | 44,2 | 99 | 3 |
| 73 | Gene_Symbol=TOP2B Isoform Beta-1 of DNA topoisomerase 2-beta | IPI00217709 | 183518 | 8,22 | 5 | 70,8 | 100 | 4 |
| 74 | Gene_Symbol=KRT16 Keratin, type I cytoskeletal 16 | IPI00217963 | 51578 | 4,99 | 4 | 92,6 | 100 | 4 |
| 75 | Gene_Symbol=DST Isoform 3 of Bullous pemphigoid antigen 1 | IPI00217992 | 308959 | 5,96 | 3 | 48,6 | 100 | 4 |
| 76 | Gene_Symbol=CACNA1E Isoform 2 of Voltage-dependent R-type calcium channel subunit alpha-1E | IPI00218338 | 261211 | 8,4 | 5 | 52,8 | 100 | 4 |
| 77 | Gene_Symbol=MLL Isoform 14P-18B of Histone-lysine N-methyltransferase HRX | IPI00218500 | 431858 | 9,27 | 5 | 64,2 | 100 | 3 |
| 78 | Gene_Symbol=TOP2A Isoform 3 of DNA topoisomerase 2-alpha | IPI00218753 | 179398 | 8,92 | 5 | 63,5 | 100 | 4 |
| 79 | Gene_Symbol=DICER1 dicer1 | IPI00219036 | 221278 | 5,47 | 3 | 51,2 | 100 | 3 |
| 80 | Gene_Symbol=PTPN2 Isoform PTPB of Tyrosine-protein phosphatase non-receptor type 2 | IPI00219695 | 48897 | 8,65 | 3 | 51,5 | 100 | 3 |
| 81 | Gene_Symbol=OGT Isoform 2 of UDP-N-acetylglucosamine--peptide N-acetylglucosaminyltransferase 110 kDa subunit | IPI00219856 | 104029 | 6,39 | 3 | 65,3 | 100 | 4 |
| 82 | Gene_Symbol=ZNF232 Isoform Short of Zinc finger protein 232 | IPI00220290 | 47339 | 6,33 | 2 | 44,7 | 99 | 4 |
| 83 | Gene_Symbol=KRT1 Keratin, type II cytoskeletal 1 | IPI00220327 | 66149 | 8,16 | 21 | 1287,4 | 100 | 4 |
| 84 | Gene_Symbol=LIMA1 Isoform Alpha of LIM domain and actin-binding protein 1 | IPI00220465 | 67534 | 5,69 | 3 | 50,0 | 100 | 3 |
| 85 | Gene_Symbol=GOLGA4 Isoform 3 of Golgin subfamily A member 4 | IPI00220521 | 261078 | 5,34 | 5 | 73,1 | 100 | 4 |
| 86 | Gene_Symbol=ITSN1 Isoform 3 of Intersectin-1 | IPI00220593 | 129450 | 7,33 | 2 | 63,8 | 100 | 3 |
| 87 | Gene_Symbol=AKAP9 A-kinase anchor protein 9 | IPI00220624 | 454925 | 4,94 | 6 | 71,1 | 100 | 4 |
| 88 | Gene_Symbol=CPO Carboxypeptidase O | IPI00220711 | 43015 | 6,6 | 1 | 42,3 | 98 | 4 |
| 89 | Gene_Symbol=DNMT1 Isoform 2 of DNA (cytosine-5)-methyltransferase 1 | IPI00220918 | 187041 | 8,13 | 6 | 74,9 | 100 | 3 |
| 90 | Gene_Symbol=IRGM Immunity-related GTPase family M protein | IPI00235330 | 20243 | 5,23 | 1 | 40,8 | 97 | 4 |
| 91 | Gene_Symbol=KIAA2022 Uncharacterized protein KIAA2022 | IPI00238220 | 169156 | 6,03 | 2 | 40,9 | 97 | 4 |
| 92 | 48 kDa protein | IPI00240503 | 48750 | 4,92 | 2 | 57,2 | 100 | 4 |
| 93 | Gene_Symbol=SLK Isoform 2 of STE20-like serine/threonine-protein kinase | IPI00247439 | 139537 | 5,03 | 3 | 62,3 | 100 | 4 |
| 94 | Gene_Symbol=FYTTD1 Forty-two-three domain-containing protein 1 | IPI00289907 | 35834 | 11,75 | 4 | 74,8 | 100 | 3 |
| 95 | Gene_Symbol=BRWD1 Isoform A of Bromodomain and WD repeat-containing protein 1 | IPI00289944 | 265493 | 8,73 | 2 | 46,6 | 99 | 3 |
| 96 | Gene_Symbol=KRT4 keratin 4 | IPI00290078 | 64442 | 8,51 | 3 | 147,3 | 100 | 3 |
| 97 | Gene_Symbol=NPAT Protein NPAT | IPI00290547 | 155507 | 5,6 | 3 | 50,8 | 100 | 4 |
| 98 | Gene_Symbol=TJP2 Tight junction protein 2 | IPI00291668 | 111993 | 7,07 | 3 | 41,5 | 98 | 4 |
| 99 | Gene_Symbol=NUP210 Isoform 1 of Nuclear pore membrane glycoprotein 210 | IPI00291755 | 205894 | 6,33 | 2 | 42,6 | 98 | 4 |
| 100 | Gene_Symbol=RGPD5;RGPD6;RGPD4 RANBP2-like and GRIP domain-containing protein 6 | IPI00293568 | 200111 | 6,03 | 5 | 62,8 | 100 | 4 |
| 101 | Gene_Symbol=CDO1 Cysteine dioxygenase type 1 | IPI00294447 | 23185 | 6,11 | 4 | 49,8 | 100 | 4 |
| 102 | Gene_Symbol=TTLL6 tubulin tyrosine ligase-like family, member 6 isoform 1 | IPI00297940 | 102482 | 9,11 | 4 | 56,0 | 100 | 3 |
| 103 | Gene_Symbol=ST5 Isoform 1 of Suppression of tumorigenicity 5 | IPI00298518 | 127617 | 9,35 | 3 | 44,8 | 99 | 3 |
| 104 | Gene_Symbol=KRT6C Keratin, type II cytoskeletal 6C | IPI00299145 | 60273 | 8,09 | 8 | 412,5 | 100 | 3 |
| 105 | Gene_Symbol=KIF14 Kinesin-like protein KIF14 | IPI00299554 | 187743 | 8,06 | 2 | 48,0 | 99 | 4 |
| 106 | Gene_Symbol=KRT84 Keratin, type II cuticular Hb4 | IPI00300052 | 65938 | 8 | 3 | 96,0 | 100 | 3 |
| 107 | Gene_Symbol=RPS4Y1;LOC100133662 40S ribosomal protein S4, Y isoform 1 | IPI00302740 | 29665 | 10,25 | 3 | 42,8 | 98 | 3 |
| 108 | Gene_Symbol=DMD Isoform 3 of Dystrophin | IPI00304639 | 427314 | 5,66 | 3 | 49,9 | 100 | 4 |
| 109 | Gene_Symbol=KRT27 Keratin, type I cytoskeletal 27 | IPI00328103 | 50420 | 5,06 | 3 | 140,3 | 100 | 4 |
| 110 | Gene_Symbol=ZC3H18 Isoform 1 of Zinc finger CCCH domain-containing protein 18 | IPI00328929 | 106525 | 8,23 | 1 | 44,5 | 99 | 3 |
| 111 | Gene_Symbol=JARID1D Isoform 1 of Histone demethylase JARID1D | IPI00329577 | 176756 | 5,59 | 3 | 53,5 | 100 | 3 |
| 112 | Gene_Symbol=AKAP13 Isoform 1 of A-kinase anchor protein 13 | IPI00329783 | 310381 | 5,12 | 4 | 41,6 | 98 | 4 |
| 113 | Gene_Symbol=OTOP3 Otopetrin-3 | IPI00332628 | 67222 | 8,96 | 1 | 52,1 | 100 | 4 |
| 114 | Gene_Symbol=AKAP4 Isoform 2 of A-kinase anchor protein 4 | IPI00333264 | 94811 | 6,68 | 4 | 71,6 | 100 | 3 |
| 115 | Gene_Symbol=NAV3 Isoform 3 of Neuron navigator 3 | IPI00333276 | 236504 | 8,76 | 2 | 56,2 | 100 | 3 |
| 116 | Gene_Symbol=ZNF677 Zinc finger protein 677 | IPI00334201 | 69720 | 9,3 | 3 | 49,1 | 100 | 3 |
| 117 | Gene_Symbol=PKP4 Isoform Short of Plakophilin-4 | IPI00334400 | 130263 | 9,15 | 3 | 52,0 | 100 | 3 |
| 118 | Gene_Symbol=RYR1 Isoform 3 of Ryanodine receptor 1 | IPI00334799 | 569892 | 5,18 | 8 | 100,0 | 100 | 4 |
| 119 | Gene_Symbol=ZNF23 Kruppel-like zinc finger factor X31 (Fragment) | IPI00335885 | 49767 | 9,21 | 1 | 41,9 | 98 | 4 |
| 120 | Gene_Symbol=FAM120B cDNA FLJ56631 | IPI00335946 | 108100 | 5,66 | 3 | 46,7 | 99 | 4 |
| 121 | Gene_Symbol=DAAM1 Isoform 2 of Disheveled-associated activator of morphogenesis 1 | IPI00337801 | 122799 | 6,81 | 3 | 62,9 | 100 | 3 |
| 122 | Gene_Symbol=PKD1L2 Isoform 1 of Polycystic kidney disease protein 1-like 2 | IPI00373872 | 275565 | 5,52 | 3 | 55,4 | 100 | 4 |
| 123 | Gene_Symbol=TMEM57 Isoform 3 of Macoilin | IPI00373988 | 51101 | 9,22 | 2 | 65,5 | 100 | 3 |
| 124 | Gene_Symbol=TMSL2 Thymosin beta-4-like protein 2 | IPI00376164 | 5037 | 5,02 | 1 | 49,0 | 100 | 4 |
| 125 | Gene_Symbol=KRT77 keratin 77 | IPI00376379 | 62149 | 5,73 | 3 | 246,0 | 100 | 4 |
| 126 | Gene_Symbol=TCEB3B RNA polymerase II transcription factor SIII subunit A2 | IPI00376867 | 84554 | 9,76 | 2 | 42,5 | 98 | 4 |
| 127 | Gene_Symbol=SSH2 cDNA FLJ58193, highly similar to Protein phosphatase Slingshot homolog 2 | IPI00377071 | 162881 | 5,17 | 3 | 53,6 | 100 | 4 |
| 128 | Gene_Symbol=NLRC5 Isoform 4 of Protein NLRC5 | IPI00383061 | 205164 | 6 | 4 | 50,9 | 100 | 3 |
| 129 | Gene_Symbol=IGHA1;IGHV3OR16-13 SNC66 protein | IPI00383164 | 54601 | 6,22 | 4 | 169,4 | 100 | 4 |
| 130 | Gene_Symbol=HNRNPM Isoform 2 of Heterogeneous nuclear ribonucleoprotein M | IPI00383296 | 73857 | 8,94 | 3 | 40,6 | 97 | 3 |
| 131 | Gene_Symbol=GFAP Isoform 2 of Glial fibrillary acidic protein | IPI00383815 | 50372 | 5,53 | 2 | 93,0 | 100 | 4 |
| 132 | Gene_Symbol=SPAG9 Isoform 2 of C-jun-amino-terminal kinase-interacting protein 4 | IPI00384432 | 145844 | 5,08 | 2 | 41,2 | 97 | 4 |
| 133 | Gene_Symbol=SF1 Isoform 5 of Splicing factor 1 | IPI00386119 | 80797 | 9,55 | 4 | 90,1 | 100 | 4 |
| 134 | Gene_Symbol=SMARCA2 Isoform Short of Probable global transcription activator SNF2L2 | IPI00386718 | 179739 | 6,83 | 3 | 44,4 | 99 | 4 |
| 135 | Gene_Symbol=RIMS1 Isoform 5 of Regulating synaptic membrane exocytosis protein 1 | IPI00387018 | 165448 | 9,52 | 4 | 47,7 | 99 | 3 |
| 136 | Ig kappa chain V-III region B6 | IPI00387113 | 11742 | 9,34 | 1 | 64,8 | 100 | 4 |
| 137 | Ig kappa chain V-III region SIE | IPI00387115 | 11882 | 8,7 | 1 | 68,1 | 100 | 4 |
| 138 | Gene_Symbol=C6orf167 Isoform 1 of Uncharacterized protein C6orf167 | IPI00394816 | 144106 | 6,81 | 2 | 52,3 | 100 | 3 |
| 139 | Gene_Symbol=GRM8 Isoform B of Metabotropic glutamate receptor 8 | IPI00396012 | 103140 | 8,49 | 2 | 68,8 | 100 | 4 |
| 140 | Gene_Symbol=PIP5K3 FYVE finger-containing phosphoinositide kinase | IPI00396145 | 239581 | 6,27 | 3 | 48,8 | 100 | 4 |
| 141 | Gene_Symbol=SNX13 Isoform 2 of Sorting nexin-13 | IPI00396267 | 111517 | 6,32 | 4 | 47,1 | 99 | 4 |
| 142 | Gene_Symbol=RGL1 Isoform A of Ral guanine nucleotide dissociation stimulator-like 1 | IPI00396431 | 87616 | 5,78 | 2 | 42,6 | 98 | 4 |
| 143 | Gene_Symbol=RNASE2 zinc finger protein 749 | IPI00397740 | 92701 | 9,01 | 2 | 47,4 | 99 | 4 |
| 144 | Gene_Symbol=PLEC1 Isoform 3 of Plectin-1 | IPI00398002 | 519655 | 5,59 | 5 | 45,5 | 99 | 4 |
| 145 | Gene_Symbol=ZNF573 Isoform 1 of Zinc finger protein 573 | IPI00398310 | 80412 | 9,32 | 3 | 40,5 | 97 | 3 |
| 146 | Gene_Symbol=CTAGE1 Protein cTAGE-2 | IPI00400827 | 85571 | 5,85 | 2 | 43,9 | 99 | 4 |
| 147 | Gene_Symbol=FLJ27365 Novel transcript | IPI00400897 | 17394 | 11,3 | 2 | 42,8 | 98 | 3 |
| 148 | Gene_Symbol=SCRN2 Ses2 protein | IPI00402502 | 41725 | 5,8 | 2 | 43,2 | 98 | 4 |
| 149 | Gene_Symbol=DNHD2 Isoform 1 of Dynein heavy chain domain-containing protein 2 | IPI00412106 | 126398 | 5,94 | 3 | 41,9 | 98 | 4 |
| 150 | Gene_Symbol=TRPM2 Isoform 2 of Transient receptor potential cation channel subfamily M member 2 | IPI00412399 | 166764 | 7,27 | 3 | 43,9 | 99 | 4 |
| 151 | Gene_Symbol=ZNF789 Isoform 1 of Zinc finger protein 789 | IPI00412697 | 51263 | 9,3 | 3 | 41,5 | 98 | 4 |
| 152 | Gene_Symbol=ARHGEF2 Isoform 2 of Rho guanine nucleotide exchange factor 2 | IPI00412782 | 112315 | 6,89 | 3 | 68,0 | 100 | 3 |
| 153 | Gene_Symbol=NBEAL1 Isoform 2 of Neurobeachin-like protein 1 | IPI00414668 | 161952 | 5,46 | 3 | 41,0 | 97 | 4 |
| 154 | Gene_Symbol=MYO1B Isoform 2 of Myosin-Ib | IPI00414980 | 125898 | 9,27 | 3 | 48,3 | 100 | 3 |
| 155 | Gene_Symbol=C4B complement component 4B preproprotein | IPI00418163 | 194169 | 6,89 | 3 | 45,2 | 99 | 4 |
| 156 | Gene_Symbol=KRT28 Keratin 25D | IPI00418663 | 53733 | 5,33 | 2 | 75,2 | 100 | 4 |
| 157 | Gene_Symbol=LOC100133739 Putative uncharacterized protein DKFZp686C15213 | IPI00426051 | 51864 | 7,85 | 3 | 185,7 | 100 | 3 |
| 158 | Gene_Symbol=IGKC Immunoblobulin light chain | IPI00430808 | 24300 | 8,29 | 4 | 333,3 | 100 | 4 |
| 159 | Gene_Symbol=FHDC1 FH2 domain-containing protein 1 | IPI00434629 | 125654 | 9,17 | 3 | 44,6 | 99 | 3 |
| 160 | Gene_Symbol=GATAD2A Isoform 2 of Transcriptional repressor p66-alpha | IPI00444526 | 65527 | 9,82 | 3 | 47,4 | 99 | 3 |
| 161 | Gene_Symbol=NCAPD2 Chromosome condensation-related SMC-associated protein 1 | IPI00445741 | 105967 | 5,78 | 2 | 42,0 | 98 | 4 |
| 162 | Gene_Symbol=SLMAP Isoform 6 of Sarcolemmal membrane-associated protein | IPI00446339 | 58008 | 5,23 | 2 | 50,9 | 100 | 4 |
| 163 | Gene_Symbol=ANKRD12 Isoform 2 of Ankyrin repeat domain-containing protein 12 | IPI00448466 | 234408 | 6,63 | 6 | 69,6 | 100 | 3 |
| 164 | Gene_Symbol=MARK4 MARK4 protein | IPI00450789 | 64302 | 9,48 | 3 | 60,7 | 100 | 3 |
| 165 | Gene_Symbol=DZIP1 Isoform 2 of Zinc finger protein DZIP1 | IPI00452092 | 97322 | 5,84 | 4 | 47,6 | 99 | 4 |
| 166 | Gene_Symbol=KIF16B Isoform 2 of Kinesin-like protein KIF16B | IPI00452247 | 160588 | 5,52 | 3 | 40,5 | 97 | 4 |
| 167 | Gene_Symbol=BRPF1 bromodomain and PHD finger-containing protein 1 isoform 1 | IPI00456620 | 139752 | 7,84 | 3 | 40,6 | 97 | 4 |
| 168 | Gene_Symbol=EIF2AK4 Isoform 2 of Eukaryotic translation initiation factor 2-alpha kinase 4 | IPI00456685 | 184899 | 6,03 | 2 | 63,9 | 100 | 4 |
| 169 | Gene_Symbol=DYNC1H1 Cytoplasmic dynein 1 heavy chain 1 | IPI00456969 | 534809 | 6,01 | 4 | 48,8 | 100 | 4 |
| 170 | Gene_Symbol=C2orf16 Uncharacterized protein C2orf16 | IPI00470912 | 225765 | 10,09 | 3 | 46,5 | 99 | 4 |
| 171 | Gene_Symbol=ANK3 Ankyrin-3 | IPI00472779 | 482387 | 6,12 | 6 | 68,5 | 100 | 3 |
| 172 | Gene_Symbol=ZNF484 Zinc finger protein 484, isoform CRA_b | IPI00477021 | 96693 | 8,74 | 3 | 45,2 | 99 | 3 |
| 173 | Gene_Symbol=SLC26A6 Anchor protein | IPI00477896 | 458487 | 6,37 | 4 | 40,6 | 97 | 4 |
| 174 | Gene_Symbol=A2M Alpha-2-macroglobulin | IPI00478003 | 164600 | 6 | 8 | 174,9 | 100 | 4 |
| 175 | Gene_Symbol=CALML4 Isoform 1 of Calmodulin-like protein 4 | IPI00478437 | 21983 | 7,02 | 2 | 40,8 | 97 | 4 |
| 176 | Gene_Symbol=ISLR2 Immunoglobulin superfamily containing leucine-rich repeat protein 2 | IPI00479654 | 79911 | 5,17 | 4 | 51,3 | 100 | 4 |
| 177 | Gene_Symbol=IGHM IGHM protein | IPI00479708 | 69309 | 6,86 | 4 | 99,9 | 100 | 4 |
| 178 | Gene_Symbol=POTEE Isoform 1 of ANKRD26-like family C member 1A | IPI00479743 | 122882 | 5,83 | 4 | 77,5 | 100 | 4 |
| 179 | Gene_Symbol=LAMA2 Putative uncharacterized protein LAMA2 | IPI00479834 | 353076 | 6,01 | 2 | 44,5 | 99 | 3 |
| 180 | Gene_Symbol=DAAM2 Disheveled-associated activator of morphogenesis 2 | IPI00514893 | 124276 | 6,36 | 3 | 55,1 | 100 | 4 |
| 181 | Gene_Symbol=SRCAP Isoform 3 of Helicase SRCAP | IPI00550342 | 329289 | 5,56 | 4 | 50,1 | 100 | 4 |
| 182 | Gene_Symbol=MORF4L1 cDNA FLJ38504 fis, clone HCHON2000156, highly similar to Mortality factor 4-like protein 1 | IPI00550968 | 40118 | 9,14 | 3 | 44,7 | 99 | 4 |
| 183 | Gene_Symbol=RBM20 RNA binding protein | IPI00552787 | 135414 | 5,48 | 2 | 52,2 | 100 | 4 |
| 184 | Gene_Symbol=SERPINA1 Isoform 1 of Alpha-1-antitrypsin | IPI00553177 | 46878 | 5,37 | 4 | 75,0 | 100 | 4 |
| 185 | Gene_Symbol=KRT8 Keratin, type II cytoskeletal 8 | IPI00554648 | 53671 | 5,52 | 5 | 268,4 | 100 | 3 |
| 186 | Gene_Symbol=GC Vitamin D-binding protein | IPI00555812 | 54526 | 5,4 | 10 | 370,2 | 100 | 4 |
| 187 | Gene_Symbol=ACTBL3 Kappa-actin | IPI00555900 | 42331 | 5,91 | 2 | 66,7 | 100 | 4 |
| 188 | Gene_Symbol=SMG1 Isoform 1 of Serine/threonine-protein kinase SMG1 | IPI00556369 | 414475 | 6,03 | 3 | 49,8 | 100 | 4 |
| 189 | Gene_Symbol=SERPING1 Serine/cysteine proteinase inhibitor clade G member 1 splice variant 2 | IPI00556459 | 37378 | 7,9 | 3 | 41,5 | 98 | 3 |
| 190 | Gene_Symbol=EXO1 Isoform 2 of Exonuclease 1 | IPI00556476 | 90201 | 8,69 | 3 | 41,6 | 98 | 4 |
| 191 | Gene_Symbol=HPR Isoform 2 of Haptoglobin-related protein | IPI00607707 | 44054 | 6,45 | 5 | 197,8 | 100 | 4 |
| 192 | Gene_Symbol=ZNF638 Isoform 3 of Zinc finger protein 638 | IPI00607784 | 219422 | 6,05 | 4 | 44,9 | 99 | 3 |
| 193 | Gene_Symbol=CENPE Isoform 3 of Centromeric protein E | IPI00619925 | 302971 | 5,45 | 6 | 74,3 | 100 | 3 |
| 194 | Gene_Symbol=PRAMEF16 PRAME family member 16 | IPI00642000 | 55943 | 8,5 | 3 | 44,6 | 99 | 3 |
| 195 | Gene_Symbol=MYH7B Isoform 1 of Myosin-7B | IPI00642716 | 222391 | 5,73 | 3 | 58,1 | 100 | 4 |
| 196 | Gene_Symbol=PHF15 PHD finger protein 15, isoform CRA_a | IPI00644322 | 95094 | 5,44 | 2 | 43,2 | 98 | 4 |
| 197 | Gene_Symbol=ZNF720 Isoform 1 of Putative protein ZNF720 | IPI00644370 | 64939 | 9,5 | 4 | 52,9 | 100 | 3 |
| 198 | Gene_Symbol=DHX32 Isoform 1 of Putative pre-mRNA-splicing factor ATP-dependent RNA helicase DHX32 | IPI00644447 | 85789 | 4,88 | 2 | 43,2 | 98 | 4 |
| 199 | Gene_Symbol=CTNNBL1 Isoform 3 of Beta-catenin-like protein 1 | IPI00645320 | 36479 | 6,24 | 4 | 79,3 | 100 | 3 |
| 200 | Gene_Symbol=CDH3 Isoform 2 of Cadherin-3 | IPI00645614 | 87012 | 4,84 | 2 | 48,4 | 100 | 4 |
| 201 | Gene_Symbol=HYDIN2 Isoform 1 of Hydrocephalus-inducing protein homolog | IPI00647188 | 580927 | 5,74 | 5 | 58,7 | 100 | 4 |
| 202 | Gene_Symbol=MICAL1 Isoform 2 of NEDD9-interacting protein with calponin homology and LIM domains | IPI00647681 | 109511 | 6,06 | 4 | 55,3 | 100 | 4 |
| 203 | Gene_Symbol=ZNF253 Zinc finger protein 253 | IPI00655840 | 59218 | 9,32 | 3 | 54,3 | 100 | 3 |
| 204 | Gene_Symbol=C14orf145 Isoform 2 of Uncharacterized protein C14orf145 | IPI00719446 | 128564 | 6,11 | 4 | 46,7 | 99 | 3 |
| 205 | Gene_Symbol=RGPD3 hypothetical protein isoform 1 | IPI00736337 | 201026 | 6,04 | 6 | 77,1 | 100 | 3 |
| 206 | Gene_Symbol=LOC442572 similar to hCG1815881 | IPI00740038 | 49569 | 9,73 | 4 | 52,2 | 100 | 3 |
| 207 | Gene_Symbol=USH2A Isoform 3 of Usherin | IPI00741527 | 588967 | 6,36 | 4 | 51,0 | 100 | 3 |
| 208 | Gene_Symbol=BAT2L HLA-B associated transcript 2-like | IPI00741537 | 243958 | 8,55 | 5 | 59,1 | 100 | 4 |
| 209 | Gene_Symbol=LOC730429 similar to E3 ubiquitin protein ligase, HECT domain containing, 1 | IPI00741894 | 312133 | 5,75 | 3 | 43,6 | 99 | 4 |
| 210 | Gene_Symbol=OBSCN Isoform 2 of Obscurin | IPI00742748 | 879717 | 5,69 | 7 | 65,9 | 100 | 4 |
| 211 | Gene_Symbol=MYH9 FLJ00279 protein (Fragment) | IPI00742780 | 66015 | 8,97 | 4 | 62,1 | 100 | 4 |
| 212 | Gene_Symbol=ARMC3 Isoform 3 of Armadillo repeat-containing protein 3 | IPI00743000 | 97530 | 5,95 | 4 | 46,1 | 99 | 4 |
| 213 | Gene_Symbol=TRAF3 TNF receptor-associated factor 3 isoform 2 | IPI00744216 | 63145 | 8,18 | 2 | 55,6 | 100 | 3 |
| 214 | Gene_Symbol=WDR87 Isoform 1 of WD repeat-containing protein 87 | IPI00744524 | 335240 | 6,94 | 5 | 65,2 | 100 | 4 |
| 215 | Gene_Symbol=REV3L Isoform 2 of DNA polymerase zeta catalytic subunit | IPI00745230 | 347244 | 8,72 | 3 | 41,0 | 97 | 4 |
| 216 | Gene_Symbol=SYNE1 Spectrin-like protein of the nuclear envelope and Golgi | IPI00745471 | 972409 | 5,4 | 6 | 67,7 | 100 | 3 |
| 217 | Gene_Symbol=MYO7B Putative uncharacterized protein MYO7B | IPI00745581 | 269972 | 8,89 | 2 | 48,0 | 99 | 3 |
| 218 | Gene_Symbol=ALB Isoform 1 of Serum albumin | IPI00745872 | 71317 | 5,92 | 63 | 5544,5 | 100 | 4 |
| 219 | Gene_Symbol=BAZ2B bromodomain adjacent to zinc finger domain, 2B | IPI00747713 | 242076 | 6,13 | 4 | 54,7 | 100 | 4 |
| 220 | Gene_Symbol=KIAA1751 Isoform 2 of Uncharacterized protein KIAA1751 | IPI00748731 | 99945 | 5,85 | 3 | 40,5 | 97 | 4 |
| 221 | Gene_Symbol=METT5D1 Isoform 1 of Putative S-adenosyl-L-methionine-dependent methyltransferase METT5D1 | IPI00783001 | 46377 | 8,8 | 3 | 49,3 | 100 | 3 |
| 222 | Gene_Symbol=C6orf163 Uncharacterized protein C6orf163 | IPI00783612 | 38757 | 6,49 | 3 | 85,1 | 100 | 4 |
| 223 | Gene_Symbol=NUP205 Nuclear pore complex protein Nup205 | IPI00783781 | 230170 | 5,83 | 3 | 50,3 | 100 | 4 |
| 224 | Gene_Symbol=JAK1 Tyrosine-protein kinase JAK1 | IPI00784013 | 135016 | 7,48 | 5 | 53,0 | 100 | 3 |
| 225 | Gene_Symbol=CEP290 Isoform 1 of Centrosomal protein of 290 kDa | IPI00784201 | 291050 | 5,75 | 5 | 51,3 | 100 | 4 |
| 226 | Gene_Symbol=VPS13D vacuolar protein sorting 13D isoform 1 | IPI00784473 | 495370 | 6,15 | 4 | 57,3 | 100 | 4 |
| 227 | Gene_Symbol=DNAH10 Isoform 1 of Dynein heavy chain 10, axonemal | IPI00784869 | 517677 | 5,64 | 3 | 51,5 | 100 | 4 |
| 228 | Gene_Symbol=RGPD8 hypothetical protein | IPI00787969 | 184348 | 6,15 | 4 | 47,9 | 99 | 4 |
| 229 | Gene_Symbol=TBC1D23 Isoform 1 of TBC1 domain family member 23 | IPI00788879 | 79184 | 5,23 | 3 | 78,9 | 100 | 4 |
| 230 | Gene_Symbol=KPNA2 Karyopherin alpha 2 | IPI00789457 | 58182 | 5,26 | 1 | 40,8 | 97 | 4 |
| 231 | Gene_Symbol=SYCP2 Synaptonemal complex protein 2 | IPI00789850 | 117545 | 9,37 | 3 | 54,0 | 100 | 3 |
| 232 | Gene_Symbol=FLJ10357 Isoform 1 of Protein SOLO | IPI00790107 | 166254 | 5,8 | 3 | 48,9 | 100 | 3 |
| 233 | Gene_Symbol=LDB2 Isoform 2 of LIM domain-binding protein 2 | IPI00790687 | 38494 | 9,13 | 3 | 45,6 | 99 | 3 |
| 234 | Gene_Symbol=LMNB1 LMNB1 protein | IPI00790831 | 44787 | 4,99 | 2 | 42,0 | 98 | 4 |
| 235 | Gene_Symbol=DIAPH3 Isoform 6 of Protein diaphanous homolog 3 | IPI00791794 | 130166 | 6,82 | 3 | 43,1 | 98 | 3 |
| 236 | Gene_Symbol=LMOD3 Isoform 1 of Leiomodin-3 | IPI00792431 | 65045 | 5,42 | 2 | 41,5 | 98 | 4 |
| 237 | 395 kDa protein | IPI00793234 | 397763 | 9,25 | 4 | 56,3 | 100 | 4 |
| 238 | Gene_Symbol=MSLN Isoform 1 of Mesothelin | IPI00793522 | 69626 | 6,03 | 4 | 44,2 | 99 | 4 |
| 239 | Gene_Symbol=KRT72 cDNA FLJ50908, highly similar to Homo sapiens keratin protein K6irs (K6IRS2) | IPI00793641 | 45128 | 5,11 | 3 | 85,9 | 100 | 4 |
| 240 | Gene_Symbol=COL7A1 Isoform 2 of Collagen alpha-1(VII) chain | IPI00795118 | 293060 | 5,92 | 4 | 61,8 | 100 | 4 |
| 241 | 156 kDa protein | IPI00795865 | 157248 | 8,39 | 3 | 44,6 | 99 | 4 |
| 242 | Gene_Symbol=RARG 17 kDa protein | IPI00795996 | 18031 | 10,92 | 1 | 46,8 | 99 | 4 |
| 243 | Gene_Symbol=LYST Isoform 1 of Lysosomal-trafficking regulator | IPI00796450 | 434169 | 6,15 | 5 | 86,3 | 100 | 4 |
| 244 | Gene_Symbol=RPH3A Isoform 2 of Rabphilin-3A | IPI00796688 | 77193 | 8,75 | 4 | 50,2 | 100 | 3 |
| 245 | Gene_Symbol=WNK2 Isoform 3 of Serine/threonine-protein kinase WNK2 | IPI00796716 | 233545 | 5,74 | 3 | 42,7 | 98 | 3 |
| 246 | Gene_Symbol=TAOK3 cDNA FLJ31808 fis, clone NT2RI2009269, highly similar to Serine/threonine-protein kinase TAO3 | IPI00796728 | 86635 | 7,03 | 2 | 46,1 | 99 | 3 |
| 247 | Gene_Symbol=KRT5 cDNA FLJ54081, highly similar to Keratin, type II cytoskeletal 5 | IPI00796776 | 60317 | 5,95 | 6 | 281,7 | 100 | 4 |
| 248 | Gene_Symbol=KNG1 20 kDa protein | IPI00797175 | 19562 | 9,3 | 4 | 127,7 | 100 | 3 |
| 249 | Gene_Symbol=PRPSAP1 phosphoribosyl pyrophosphate synthetase-associated protein 1 | IPI00797603 | 42726 | 8,66 | 2 | 42,5 | 98 | 3 |
| 250 | Gene_Symbol=WDR6 WD repeat domain 6 protein | IPI00797612 | 126917 | 6,45 | 2 | 44,2 | 99 | 4 |
| 251 | Gene_Symbol=SPG11 Isoform 2 of Spatacsin | IPI00798078 | 239529 | 5,43 | 2 | 42,5 | 98 | 4 |
| 252 | Gene_Symbol=ZNF229 Zinc finger protein 229 | IPI00798156 | 96654 | 8,91 | 3 | 41,2 | 98 | 3 |
| 253 | Gene_Symbol=KIF19 Isoform 1 of Kinesin-like protein KIF19 | IPI00827544 | 111834 | 8,87 | 3 | 42,2 | 98 | 3 |
| 254 | Gene_Symbol=BEND5 Isoform 2 of Coiled-coil domain-containing protein Bend5 | IPI00827810 | 29425 | 6,92 | 2 | 45,2 | 99 | 4 |
| 255 | Gene_Symbol=IGL@ IGL@ protein | IPI00829626 | 25287 | 5,21 | 3 | 188,8 | 100 | 4 |
| 256 | Gene_Symbol=IGHG4 Ig gamma-4 chain C region | IPI00829814 | 36431 | 7,18 | 3 | 181,3 | 100 | 4 |
| 257 | Gene_Symbol=IGKC IGKC protein | IPI00845354 | 25715 | 6,3 | 4 | 252,7 | 100 | 4 |
| 258 | Gene_Symbol=BAHCC1 BAH and coiled-coil domain-containing protein 1 | IPI00845508 | 279554 | 9,02 | 3 | 50,6 | 100 | 3 |
| 259 | Gene_Symbol=APOA4 apolipoprotein A-IV precursor | IPI00847179 | 45344 | 5,28 | 3 | 76,2 | 100 | 3 |
| 260 | Gene_Symbol=KRT7 keratin 7 | IPI00847342 | 51411 | 5,4 | 4 | 177,6 | 100 | 4 |
| 261 | Gene_Symbol=LBA1 Lupus brain antigen 1 homolog | IPI00847543 | 340341 | 6,34 | 3 | 55,9 | 100 | 4 |
| 262 | Gene_Symbol=RIMS1 Isoform 2 of Regulating synaptic membrane exocytosis protein 1 | IPI00848003 | 188861 | 9,65 | 4 | 54,7 | 100 | 3 |
| 263 | Gene_Symbol=DIAPH1 Isoform 1 of Protein diaphanous homolog 1 | IPI00852685 | 141942 | 5,31 | 2 | 41,8 | 98 | 4 |
| 264 | Gene_Symbol=RHBDF1 cDNA FLJ60400, highly similar to Homo sapiens rhomboid family 1 (RHBDF1) | IPI00852996 | 64499 | 10,27 | 3 | 84,0 | 100 | 4 |
| 265 | Gene_Symbol=HBA1;HBA2 Alpha 2 globin variant (Fragment) | IPI00853068 | 15328 | 8,72 | 2 | 98,3 | 100 | 3 |
| 266 | Gene_Symbol=JAKMIP1 Isoform 2 of Janus kinase and microtubule-interacting protein 1 | IPI00853270 | 97718 | 5,63 | 3 | 50,1 | 100 | 4 |
| 267 | Transthyretin | IPI00855916 | 20300 | 5,16 | 3 | 184,3 | 100 | 4 |
| 268 | Gene_Symbol=MAP3K1 Mitogen-activated protein kinase kinase kinase 1 | IPI00855985 | 166419 | 7,93 | 5 | 87,1 | 100 | 4 |
| 269 | Gene_Symbol=CENPF Centromere protein F | IPI00855998 | 370843 | 5,03 | 5 | 58,1 | 100 | 4 |
| 270 | Gene_Symbol=NAV2 268 kDa protein | IPI00871234 | 269376 | 9,15 | 6 | 88,1 | 100 | 3 |
| 271 | Gene_Symbol=ZFYVE28 107 kDa protein | IPI00871556 | 109411 | 5,46 | 4 | 92,4 | 100 | 4 |
| 272 | Gene_Symbol=HDAC5 127 kDa protein | IPI00871583 | 127408 | 6,03 | 5 | 57,8 | 100 | 4 |
| 273 | Gene_Symbol=LOC646508 similar to hCG2036632 | IPI00871753 | 62046 | 9,88 | 3 | 46,1 | 99 | 3 |
| 274 | Gene_Symbol=DDX60 200 kDa protein | IPI00871808 | 202061 | 7,78 | 3 | 43,1 | 98 | 4 |
| 275 | Gene_Symbol=ZNF423 Putative uncharacterized protein ZNF423 (Fragment) | IPI00872102 | 148755 | 6,43 | 4 | 52,8 | 100 | 4 |
| 276 | Gene_Symbol=GLT25D2 84 kDa protein | IPI00872640 | 84924 | 6,51 | 3 | 43,9 | 99 | 4 |
| 277 | Gene_Symbol=MLL2 564 kDa protein | IPI00872759 | 569905 | 5,65 | 4 | 44,1 | 99 | 4 |
| 278 | Gene_Symbol=WDR19 cDNA FLJ56808, highly similar to WD repeat protein 19 | IPI00873175 | 146656 | 6,04 | 3 | 41,9 | 98 | 4 |
| 279 | Gene_Symbol=CELSR1 Putative uncharacterized protein CELSR1 | IPI00874160 | 334297 | 5,58 | 2 | 47,4 | 99 | 4 |
| 280 | Gene_Symbol=MYO18B Isoform 1 of Myosin-XVIIIb | IPI00874168 | 287175 | 6,49 | 3 | 42,2 | 98 | 4 |
| 281 | Gene_Symbol=DNAH1 Isoform 1 of Dynein heavy chain 1, axonemal | IPI00878816 | 497704 | 5,66 | 2 | 40,8 | 97 | 4 |
| 282 | Gene_Symbol=KRT73 Isoform 2 of Keratin, type II cytoskeletal 73 | IPI00878987 | 42244 | 8,42 | 2 | 111,4 | 100 | 4 |
| 283 | Gene_Symbol=TTLL12 Tubulin tyrosine ligase-like family, member 12 | IPI00879002 | 74185 | 5,34 | 3 | 44,7 | 99 | 4 |
| 284 | Gene_Symbol=ACSBG2 bubblegum related protein | IPI00879473 | 74934 | 8,67 | 3 | 57,4 | 100 | 4 |
| 285 | Gene_Symbol=ATP6AP1 | IPI00879556 | 14340 | 5,71 | 2 | 49,2 | 100 | 4 |
| 286 | Gene_Symbol=SETDB1 Isoform 3 of Histone-lysine N-methyltransferase SETDB1 | IPI00879832 | 144963 | 5,7 | 3 | 41,7 | 98 | 4 |
| 287 | 29 kDa protein | IPI00879936 | 28920 | 4,83 | 1 | 60,1 | 100 | 4 |
| 288 | Gene_Symbol=CCDC66 coiled-coil domain containing 66 isoform 1 | IPI00884155 | 110256 | 8,47 | 3 | 50,9 | 100 | 4 |
| 289 | Gene_Symbol=PATL1 Isoform 1 of Protein PAT1 homolog 1 | IPI00885108 | 87138 | 6,22 | 2 | 42,7 | 98 | 4 |
| 290 | Gene_Symbol=SLC25A25 Isoform 4 of Calcium-binding mitochondrial carrier protein SCaMC-2 | IPI00885125 | 54888 | 8,76 | 3 | 48,3 | 100 | 3 |
| 291 | Gene_Symbol=LOC653071 similar to ankyrin repeat domain 57 | IPI00886810 | 41604 | 9,79 | 4 | 59,6 | 100 | 3 |
| 292 | Gene_Symbol=LOC644548 hypothetical LOC644548 | IPI00886845 | 32628 | 6,56 | 2 | 70,1 | 100 | 3 |
| 293 | Gene_Symbol=hCG_2045206 hypothetical LOC100131655 | IPI00887123 | 169808 | 10,25 | 5 | 56,7 | 100 | 4 |
| 294 | Gene_Symbol=FRMPD3 similar to FERM and PDZ domain-containing protein 3 | IPI00887257 | 181782 | 8,34 | 3 | 41,7 | 98 | 4 |
| 295 | Gene_Symbol=LOC100134794 similar to keratin 8 | IPI00887509 | 12467 | 10,16 | 2 | 74,1 | 100 | 4 |
| 296 | Gene_Symbol=hCG_1774568 similar to hCG1774568 | IPI00887605 | 32051 | 11,81 | 1 | 43,3 | 98 | 4 |
| 297 | Gene_Symbol=GUCY2G similar to guanylyl cyclase receptor G | IPI00887656 | 123300 | 9,71 | 3 | 40,6 | 97 | 4 |
| 298 | Gene_Symbol=LOC646508 similar to hCG2036632 | IPI00887727 | 59412 | 8,47 | 5 | 63,1 | 100 | 3 |
| 299 | Gene_Symbol=LOC728965 similar to hCG1749101 | IPI00887730 | 78397 | 11,2 | 4 | 54,2 | 100 | 3 |
| 300 | Gene_Symbol=LOC100133758 hypothetical protein, partial | IPI00887746 | 53607 | 4,43 | 4 | 43,5 | 99 | 4 |
| 301 | Gene_Symbol=LOC100129958 similar to hCG1643231 | IPI00888053 | 35448 | 8,21 | 3 | 113,0 | 100 | 4 |
| 302 | Gene_Symbol=FAT4 Isoform 3 of Protocadherin Fat 4 | IPI00888207 | 546214 | 4,77 | 6 | 74,3 | 100 | 4 |
| 303 | Gene_Symbol=LOC728498 similar to golgi autoantigen, golgin subfamily a, 8A isoform 1 | IPI00888557 | 68545 | 8,92 | 2 | 41,5 | 98 | 4 |
| 304 | Gene_Symbol=ACTBL3 similar to protein expressed in prostate, ovary, testis, and placenta 2 | IPI00888712 | 106037 | 9,35 | 2 | 49,6 | 100 | 3 |
| 305 | Gene_Symbol=LOC100129520 similar to hCG2044193 | IPI00888854 | 110419 | 8,94 | 4 | 51,1 | 100 | 3 |
| 306 | Gene_Symbol=TTC13 tetratricopeptide repeat domain 13 isoform b | IPI00890709 | 91644 | 7,07 | 3 | 51,3 | 100 | 4 |
| 307 | Gene_Symbol=PION Isoform 1 of Protein pigeon homolog | IPI00893006 | 98993 | 6,38 | 3 | 43,5 | 99 | 4 |
| 308 | Gene_Symbol=RYR3 Putative uncharacterized protein RYR3 | IPI00893362 | 557631 | 5,48 | 3 | 48,9 | 100 | 4 |
| 309 | Gene_Symbol=ABCB5 ATP-binding cassette, sub-family B (MDR/TAP), member 5 | IPI00893833 | 139294 | 7,29 | 2 | 49,9 | 100 | 4 |
| 310 | Gene_Symbol=NAG Protein | IPI00894456 | 24047 | 5,79 | 1 | 47,6 | 99 | 4 |
| 311 | DMXL2 protein | IPI00896496 | 272828 | 6,12 | 2 | 45,9 | 99 | 4 |
| 312 | Gene_Symbol=HP Haptoglobin | IPI00902590 | 45861 | 6,13 | 9 | 346,3 | 100 | 4 |
| 313 | Gene_Symbol=HPR cDNA FLJ31310 fis | IPI00902867 | 31672 | 8,48 | 7 | 225,4 | 100 | 4 |
| 314 | Gene_Symbol=BAT2D1 Isoform 2 of BAT2 domain-containing protein 1 | IPI00902970 | 291556 | 9,2 | 3 | 40,5 | 97 | 4 |
| 315 | cDNA FLJ58281 | IPI00908990 | 61849 | 8,39 | 2 | 46,1 | 99 | 4 |
| 316 | cDNA FLJ54284, highly similar to Transcription initiation factor IIF alpha subunit | IPI00909065 | 46409 | 5,75 | 4 | 58,5 | 100 | 4 |
| 317 | cDNA FLJ56758, highly similar to Dolichyl-phosphate beta-glucosyltransferase | IPI00909749 | 25520 | 9,14 | 4 | 60,3 | 100 | 4 |
| 318 | cDNA FLJ58651, highly similar to Alpha-actinin-3 | IPI00910616 | 108498 | 6 | 4 | 55,8 | 100 | 4 |
| 319 | cDNA FLJ59792, highly similar to Homo sapiens outer dense fiber of sperm tails 2-like (ODF2L) | IPI00910887 | 68103 | 6,19 | 2 | 52,3 | 100 | 4 |
| 320 | Gene_Symbol=NEB Nebulin | IPI00914847 | 775749 | 9,1 | 8 | 79,7 | 100 | 4 |
| 321 | Gene_Symbol=USP54 Isoform 4 of Inactive ubiquitin carboxyl-terminal hydrolase 54 | IPI00914881 | 172243 | 7,35 | 2 | 42,8 | 98 | 4 |
| 322 | 521 kDa protein | IPI00915428 | 524926 | 6,03 | 5 | 41,4 | 98 | 4 |
| 323 | Gene_Symbol=TTC28 Tetratricopeptide repeat protein 28 | IPI00915468 | 233452 | 6,24 | 3 | 41,2 | 98 | 4 |
| 324 | Gene_Symbol=MLL3 Isoform 3 of Histone-lysine N-methyltransferase MLL3 | IPI00916332 | 554529 | 6,08 | 5 | 47,7 | 99 | 4 |
| 325 | Gene_Symbol=SETD2 Protein | IPI00917578 | 147589 | 5,88 | 4 | 53,6 | 100 | 4 |
| 326 | Gene_Symbol=EFTUD2 elongation factor Tu GTP binding domain containing 2 isoform b | IPI00917777 | 106286 | 5,06 | 3 | 47,7 | 99 | 4 |
