# Supplementary material for: An automated plasma protein fractionation design: high-throughput perspectives for proteomic analysis
Source: BMC Res Notes. 2012 Nov 1;5:612. doi: 10.1186/1756-0500-5-612 (PMC3517536; doi:10.1186/1756-0500-5-612)
Supplement: Additional file 4 — Supporting Table 2. Proteins identified in the basic fraction. Protein name, accession number, pI and other additional information are reported. [file 1756-0500-5-612-S4.doc]

| **N** | **Protein Name** | **Accession Number** | **MW** | **PI** | **Peptide Count** | **Total Ion Score** | **Total Ion Score C.I. %** | **N tech replicates** |
| --- | --- | --- | --- | --- | --- | --- | --- | --- |
| 1 | Gene_Symbol=WASF4 Wiskott-Aldrich syndrome protein family member 4 | IPI00002647 | 68602 | 6,26 | 3 | 41,0 | 98 | 3 |
| 2 | Gene_Symbol=KRT24 Keratin, type I cytoskeletal 24 | IPI00004550 | 55567 | 4,89 | 3 | 78,2 | 100 | 4 |
| 3 | Gene_Symbol=MRPS12 28S ribosomal protein S12, mitochondrial | IPI00005692 | 15562 | 10,32 | 2 | 40,9 | 98 | 4 |
| 4 | Gene_Symbol=HERC2 Probable E3 ubiquitin-protein ligase HERC2 | IPI00005826 | 533697 | 5,86 | 2 | 40,4 | 97 | 4 |
| 5 | Gene_Symbol=KRT75 cDNA FLJ60809, cytokeratin type II (K6HF) | IPI00005859 | 65720 | 8,52 | 5 | 200,0 | 100 | 4 |
| 6 | Gene_Symbol=CEP164 Isoform 1 of Centrosomal protein of 164 kDa | IPI00007293 | 164727 | 5,27 | 3 | 70,7 | 100 | 4 |
| 7 | Gene_Symbol=KRT10 Keratin, type I cytoskeletal 10 | IPI00009865 | 59703 | 5,13 | 16 | 689,9 | 100 | 3 |
| 8 | Gene_Symbol=APC Isoform Long of Adenomatous polyposis coli protein | IPI00012391 | 313620 | 7,92 | 7 | 60,2 | 100 | 4 |
| 9 | Gene_Symbol=KRT12 Keratin, type I cytoskeletal 12 | IPI00015309 | 53592 | 4,7 | 3 | 92,3 | 100 | 3 |
| 10 | Gene_Symbol=TCHH Trichohyalin | IPI00015869 | 254233 | 5,73 | 5 | 42,5 | 98 | 3 |
| 11 | Gene_Symbol=RHOH Rho-related GTP-binding protein RhoH | IPI00018882 | 21716 | 9,17 | 2 | 41,6 | 98 | 4 |
| 12 | Gene_Symbol=KRT9 Keratin, type I cytoskeletal 9 | IPI00019359 | 62320 | 5,19 | 14 | 444,0 | 100 | 3 |
| 13 | Gene_Symbol=MYH9 Isoform 1 of Myosin-9 | IPI00019502 | 227646 | 5,5 | 6 | 76,1 | 100 | 4 |
| 14 | Gene_Symbol=BSN Protein bassoon | IPI00020153 | 418354 | 7,28 | 4 | 45,1 | 99 | 4 |
| 15 | Gene_Symbol=KRT2 Keratin, type II cytoskeletal 2 epidermal | IPI00021304 | 66111 | 8,07 | 13 | 516,7 | 100 | 4 |
| 16 | Gene_Symbol=KIF13B Kinesin-like protein KIF13B | IPI00021753 | 203907 | 5,56 | 4 | 60,5 | 100 | 4 |
| 17 | Gene_Symbol=HRG Histidine-rich glycoprotein | IPI00022371 | 60510 | 7,09 | 9 | 320,4 | 100 | 3 |
| 18 | Gene_Symbol=TF Serotransferrin | IPI00022463 | 79280 | 6,81 | 26 | 1771,9 | 100 | 4 |
| 19 | Gene_Symbol=HPX Hemopexin | IPI00022488 | 52385 | 6,55 | 5 | 178,8 | 100 | 4 |
| 20 | Gene_Symbol=PIK3R4 Phosphoinositide 3-kinase regulatory subunit 4 | IPI00024006 | 154318 | 6,74 | 2 | 48,8 | 100 | 3 |
| 21 | Gene_Symbol=APC2 Isoform 1 of Adenomatous polyposis coli protein 2 | IPI00025190 | 245966 | 9,08 | 3 | 40,1 | 97 | 4 |
| 22 | Gene_Symbol=FAT1 Protocadherin Fat 1 | IPI00031411 | 509685 | 4,85 | 3 | 72,1 | 100 | 4 |
| 23 | Gene_Symbol=KNG1 Isoform HMW of Kininogen-1 | IPI00032328 | 72996 | 6,34 | 3 | 83,1 | 100 | 3 |
| 24 | Gene_Symbol=DNM2 Isoform 1 of Dynamin-2 | IPI00033022 | 98345 | 7,04 | 2 | 51,2 | 100 | 4 |
| 25 | Gene_Symbol=ZNF407 Isoform 1 of Zinc finger protein 407 | IPI00043516 | 251658 | 6,05 | 4 | 53,1 | 100 | 3 |
| 26 | Gene_Symbol=TGM7 Protein-glutamine gamma-glutamyltransferase Z | IPI00044409 | 80575 | 6,54 | 2 | 51,7 | 100 | 4 |
| 27 | Gene_Symbol=SPEN Msx2-interacting protein | IPI00045914 | 403030 | 7,35 | 3 | 42,3 | 98 | 4 |
| 28 | Gene_Symbol=SPG11 Isoform 1 of Spatacsin | IPI00101923 | 282621 | 5,63 | 2 | 44,0 | 99 | 3 |
| 29 | Gene_Symbol=SSH1 Isoform 3 of Protein phosphatase Slingshot homolog 1 | IPI00103741 | 105975 | 5,83 | 2 | 43,8 | 99 | 4 |
| 30 | Gene_Symbol=DNAH3 Isoform 1 of Dynein heavy chain 3, axonemal | IPI00152462 | 473776 | 6,04 | 3 | 58,8 | 100 | 3 |
| 31 | Gene_Symbol=DNAH5 Dynein heavy chain 5, axonemal | IPI00152653 | 532504 | 5,79 | 4 | 59,4 | 100 | 3 |
| 32 | Gene_Symbol=IGHA1;IGHV3OR16-13 IGHA1 protein | IPI00166866 | 54483 | 6,26 | 3 | 130,0 | 100 | 3 |
| 33 | Gene_Symbol=EFCAB3 EF-hand calcium-binding domain-containing protein 3 | IPI00167171 | 50685 | 9,31 | 2 | 40,3 | 97 | 4 |
| 34 | Gene_Symbol=ZNF676 Zinc finger protein 676 | IPI00167265 | 93840 | 9,1 | 4 | 48,0 | 100 | 4 |
| 35 | Gene_Symbol=XIRP2 Isoform 4 of Xin actin-binding repeat-containing protein 2 | IPI00167400 | 107728 | 5,95 | 3 | 40,9 | 98 | 4 |
| 36 | Gene_Symbol=ATAD2 Isoform 1 of ATPase family AAA domain-containing protein 2 | IPI00170548 | 159825 | 5,94 | 3 | 51,4 | 100 | 3 |
| 37 | Gene_Symbol=KRT73 Isoform 1 of Keratin, type II cytoskeletal 73 | IPI00174775 | 59457 | 6,93 | 4 | 126,3 | 100 | 4 |
| 38 | Gene_Symbol=NPHP4 Nephrocystin-4 | IPI00176920 | 158754 | 8,4 | 2 | 43,6 | 99 | 4 |
| 39 | Gene_Symbol=ZNF638 Isoform 4 of Zinc finger protein 638 | IPI00178953 | 129378 | 9,57 | 3 | 42,7 | 98 | 4 |
| 40 | Gene_Symbol=DNAH7 Isoform 1 of Dynein heavy chain 7, axonemal | IPI00180384 | 464382 | 5,7 | 2 | 42,5 | 98 | 3 |
| 41 | Gene_Symbol=DNMT3B Isoform 6 of DNA (cytosine-5)-methyltransferase 3B | IPI00180702 | 96054 | 8,03 | 4 | 61,1 | 100 | 3 |
| 42 | Gene_Symbol=MYO3A Myosin IIIA | IPI00185036 | 187574 | 9,04 | 3 | 41,4 | 98 | 4 |
| 43 | Gene_Symbol=OTOF Isoform 3 of Otoferlin | IPI00216364 | 149989 | 5,72 | 2 | 47,7 | 100 | 3 |
| 44 | Gene_Symbol=KRT16 Keratin, type I cytoskeletal 16 | IPI00217963 | 51578 | 4,99 | 4 | 108,1 | 100 | 3 |
| 45 | Gene_Symbol=MINK1 Isoform 1 of Misshapen-like kinase 1 | IPI00218497 | 146388 | 6,95 | 2 | 45,6 | 99 | 3 |
| 46 | Gene_Symbol=KRT1 Keratin, type II cytoskeletal 1 | IPI00220327 | 66149 | 8,16 | 20 | 1254,3 | 100 | 3 |
| 47 | Gene_Symbol=LIMA1 Isoform Alpha of LIM domain and actin-binding protein 1 | IPI00220465 | 67534 | 5,69 | 3 | 54,3 | 100 | 3 |
| 48 | Gene_Symbol=GOLGA4 Isoform 3 of Golgin subfamily A member 4 | IPI00220521 | 261078 | 5,34 | 3 | 61,0 | 100 | 4 |
| 49 | Gene_Symbol=AKAP9 A-kinase anchor protein 9 | IPI00220624 | 454925 | 4,94 | 4 | 52,6 | 100 | 4 |
| 50 | Gene_Symbol=SYNE2 Isoform 2 of Nesprin-2 | IPI00239406 | 804235 | 5,25 | 5 | 53,0 | 100 | 3 |
| 51 | Gene_Symbol=ELFN2 Leucine-rich repeat and fibronectin type-III domain-containing protein 6 | IPI00289849 | 90714 | 7,56 | 3 | 50,7 | 100 | 4 |
| 52 | Gene_Symbol=KRT3 Keratin, type II cytoskeletal 3 | IPI00290857 | 64636 | 6,12 | 5 | 202,7 | 100 | 4 |
| 53 | Gene_Symbol=NDUFV3 NADH-ubiquinone oxidoreductase flavoprotein 3 isoform a precursor | IPI00291016 | 51010 | 9,18 | 5 | 53,6 | 100 | 4 |
| 54 | Gene_Symbol=NUMA1 Isoform 1 of Nuclear mitotic apparatus protein 1 | IPI00292771 | 239199 | 5,63 | 4 | 83,1 | 100 | 4 |
| 55 | Gene_Symbol=ZC3H18 Isoform 2 of Zinc finger CCCH domain-containing protein 18 | IPI00293312 | 84505 | 5,47 | 4 | 91,4 | 100 | 3 |
| 56 | Gene_Symbol=SMARCA5 SWI/SNF-related matrix-associated actin-dependent regulator of chromatin subfamily A member 5 | IPI00297211 | 122513 | 8,27 | 4 | 47,4 | 99 | 4 |
| 57 | Gene_Symbol=IRF7 Isoform B of Interferon regulatory factor 7 | IPI00298039 | 52225 | 6,11 | 2 | 73,5 | 100 | 4 |
| 58 | Gene_Symbol=KRT6C Keratin, type II cytoskeletal 6C | IPI00299145 | 60273 | 8,09 | 10 | 478,0 | 100 | 4 |
| 59 | Gene_Symbol=KIF14 Kinesin-like protein KIF14 | IPI00299554 | 187743 | 8,06 | 3 | 48,3 | 100 | 4 |
| 60 | Gene_Symbol=KRT84 Keratin, type II cuticular Hb4 | IPI00300052 | 65938 | 8 | 2 | 94,7 | 100 | 4 |
| 61 | Gene_Symbol=KRT82 Keratin, type II cuticular Hb2 | IPI00300053 | 57985 | 6,4 | 3 | 107,5 | 100 | 3 |
| 62 | Gene_Symbol=TRNT1 Isoform 2 of tRNA-nucleotidyltransferase 1, mitochondrial | IPI00301719 | 48043 | 8,61 | 1 | 42,0 | 98 | 4 |
| 63 | Gene_Symbol=CHRD Isoform 1 of Chordin | IPI00306710 | 104703 | 8,07 | 1 | 44,1 | 99 | 4 |
| 64 | Gene_Symbol=KRT27 Keratin, type I cytoskeletal 27 | IPI00328103 | 50420 | 5,06 | 3 | 138,9 | 100 | 3 |
| 65 | Gene_Symbol=OTOP3 Otopetrin-3 | IPI00332628 | 67222 | 8,96 | 1 | 52,1 | 100 | 4 |
| 66 | Gene_Symbol=GCC2 Isoform 2 of GRIP and coiled-coil domain-containing protein 2 | IPI00333197 | 196872 | 5,1 | 2 | 44,2 | 99 | 3 |
| 67 | Gene_Symbol=PKP4 Isoform Short of Plakophilin-4 | IPI00334400 | 130263 | 9,15 | 3 | 52,0 | 100 | 4 |
| 68 | Gene_Symbol=RYR1 Isoform 3 of Ryanodine receptor 1 | IPI00334799 | 569892 | 5,18 | 5 | 84,0 | 100 | 4 |
| 69 | Gene_Symbol=KTN1 Isoform 2 of Kinectin | IPI00337736 | 149804 | 5,57 | 2 | 46,3 | 99 | 3 |
| 70 | Gene_Symbol=HMMR hyaluronan-mediated motility receptor isoform a | IPI00337772 | 84576 | 5,73 | 2 | 39,9 | 97 | 4 |
| 71 | Gene_Symbol=PCNX Isoform 3 of Pecanex-like protein 1 | IPI00375256 | 253129 | 6,98 | 3 | 42,3 | 98 | 3 |
| 72 | Gene_Symbol=PRKDC Isoform 2 of DNA-dependent protein kinase catalytic subunit | IPI00376215 | 470162 | 6,81 | 3 | 55,5 | 100 | 4 |
| 73 | Gene_Symbol=KRT77 keratin 77 | IPI00376379 | 62149 | 5,73 | 3 | 258,9 | 100 | 3 |
| 74 | Gene_Symbol=SSH2 Protein phosphatase Slingshot homolog 2 | IPI00377071 | 162881 | 5,17 | 3 | 65,7 | 100 | 4 |
| 75 | Gene_Symbol=NEDD4 neural precursor cell expressed, developmentally down-regulated 4 isoform 2 | IPI00384495 | 141862 | 6,19 | 3 | 45,7 | 99 | 3 |
| 76 | Gene_Symbol=SF1 Isoform 5 of Splicing factor 1 | IPI00386119 | 80797 | 9,55 | 3 | 59,9 | 100 | 4 |
| 77 | Gene_Symbol=- Ig kappa chain V-III region B6 | IPI00387113 | 11742 | 9,34 | 1 | 64,8 | 100 | 4 |
| 78 | Gene_Symbol=- Ig kappa chain V-III region SIE | IPI00387115 | 11882 | 8,7 | 1 | 68,1 | 100 | 4 |
| 79 | Gene_Symbol=GRM8 Isoform B of Metabotropic glutamate receptor 8 | IPI00396012 | 103140 | 8,49 | 2 | 64,0 | 100 | 4 |
| 80 | Gene_Symbol=ATXN7L1 ataxin 7-like 1 isoform 3 | IPI00397389 | 95398 | 9,65 | 3 | 41,7 | 98 | 4 |
| 81 | Gene_Symbol=ZNF844 Zinc finger protein 844 | IPI00397691 | 78708 | 9,14 | 2 | 54,0 | 100 | 4 |
| 82 | Gene_Symbol=RNASE2 zinc finger protein 749 | IPI00397740 | 92701 | 9,01 | 2 | 59,9 | 100 | 4 |
| 83 | Gene_Symbol=PLEC1 Isoform 3 of Plectin-1 | IPI00398002 | 519655 | 5,59 | 6 | 63,9 | 100 | 4 |
| 84 | Gene_Symbol=ZDHHC17 Isoform 1 of Palmitoyltransferase ZDHHC17 | IPI00410687 | 73732 | 7,29 | 2 | 57,2 | 100 | 4 |
| 85 | Gene_Symbol=OSGIN1 Isoform 1 of Oxidative stress-induced growth inhibitor 1 | IPI00413139 | 52188 | 6,63 | 3 | 53,1 | 100 | 3 |
| 86 | Gene_Symbol=C4B complement component 4B preproprotein | IPI00418163 | 194170 | 6,89 | 4 | 60,1 | 100 | 4 |
| 87 | Gene_Symbol=OPLAH 5-oxoprolinase | IPI00418382 | 138739 | 6,12 | 3 | 47,9 | 100 | 3 |
| 88 | Gene_Symbol=LOC100133739 Putative uncharacterized protein DKFZp686C15213 | IPI00426051 | 51864 | 7,85 | 4 | 197,2 | 100 | 4 |
| 89 | Gene_Symbol=IGKC Immunoblobulin light chain (Fragment) | IPI00430808 | 24300 | 8,29 | 4 | 332,8 | 100 | 4 |
| 90 | Gene_Symbol=MCTP1 Isoform 1 of Multiple C2 and transmembrane domain-containing protein 1 | IPI00431791 | 112403 | 8,48 | 3 | 41,1 | 98 | 4 |
| 91 | Gene_Symbol=KRT17 Keratin, type I cytoskeletal 17 | IPI00450768 | 48361 | 4,97 | 3 | 81,3 | 100 | 3 |
| 92 | Gene_Symbol=MARK4 MARK4 protein | IPI00450789 | 64302 | 9,48 | 2 | 43,5 | 99 | 4 |
| 93 | Gene_Symbol=ITSN1 Intersectin 1 short form transcript variant 8 | IPI00451615 | 116071 | 8,64 | 2 | 46,0 | 99 | 4 |
| 94 | Gene_Symbol=GALNT11 Isoform 1 of Polypeptide N-acetylgalactosaminyltransferase 11 | IPI00456589 | 69730 | 8,47 | 3 | 43,5 | 99 | 3 |
| 95 | Gene_Symbol=EIF2AK4 Isoform 2 of Eukaryotic translation initiation factor 2-alpha kinase 4 | IPI00456685 | 184899 | 6,03 | 2 | 53,2 | 100 | 4 |
| 96 | Gene_Symbol=SH3PXD2A Isoform 2 of SH3 and PX domain-containing protein 2A | IPI00456943 | 103069 | 8,09 | 3 | 39,9 | 97 | 3 |
| 97 | Gene_Symbol=DYNC1H1 Cytoplasmic dynein 1 heavy chain 1 | IPI00456969 | 534809 | 6,01 | 5 | 92,9 | 100 | 4 |
| 98 | Gene_Symbol=SIK2 Serine/threonine-protein kinase SNF1-like kinase 2 | IPI00465291 | 104705 | 5,7 | 2 | 40,3 | 97 | 3 |
| 99 | Gene_Symbol=NCOA1 Nuclear receptor coactivator 1 isoform 2 | IPI00470491 | 155021 | 5,84 | 3 | 40,3 | 97 | 3 |
| 100 | Gene_Symbol=CEP152 Isoform 2 of Centrosomal protein of 152 kDa | IPI00477050 | 197899 | 5,45 | 3 | 41,4 | 98 | 3 |
| 101 | Gene_Symbol=ADAM23 Isoform Gamma of ADAM 23 | IPI00477723 | 94637 | 6,84 | 3 | 55,2 | 100 | 3 |
| 102 | Gene_Symbol=FREM1 Isoform 1 of FRAS1-related extracellular matrix protein 1 | IPI00477820 | 245953 | 5,56 | 3 | 40,5 | 97 | 3 |
| 103 | Gene_Symbol=SLC26A6 Anchor protein | IPI00477896 | 458487 | 6,37 | 4 | 45,7 | 99 | 4 |
| 104 | Gene_Symbol=A2M Alpha-2-macroglobulin | IPI00478003 | 164600 | 6 | 4 | 71,4 | 100 | 4 |
| 105 | Gene_Symbol=KIF15 Isoform 4 of Kinesin-like protein KIF15 | IPI00479255 | 137939 | 5,8 | 3 | 53,3 | 100 | 3 |
| 106 | Gene_Symbol=IGHM IGHM protein | IPI00479708 | 69309 | 6,86 | 3 | 81,9 | 100 | 4 |
| 107 | Gene_Symbol=POTEE Isoform 1 of ANKRD26-like family C member 1A | IPI00479743 | 122882 | 5,83 | 2 | 56,0 | 100 | 4 |
| 108 | Gene_Symbol=DAAM2 Disheveled-associated activator of morphogenesis 2 | IPI00514893 | 124276 | 6,36 | 4 | 93,9 | 100 | 4 |
| 109 | Gene_Symbol=CEP135 Isoform 1 of Centrosomal protein of 135 kDa | IPI00550987 | 133878 | 5,87 | 3 | 45,9 | 99 | 4 |
| 110 | Gene_Symbol=SERPINA1 Isoform 1 of Alpha-1-antitrypsin | IPI00553177 | 46878 | 5,37 | 3 | 61,2 | 100 | 4 |
| 111 | Gene_Symbol=ATR Isoform 2 of Serine/threonine-protein kinase ATR | IPI00554573 | 297451 | 7,16 | 3 | 40,4 | 97 | 4 |
| 112 | Gene_Symbol=KRT8 Keratin, type II cytoskeletal 8 | IPI00554648 | 53671 | 5,52 | 3 | 200,1 | 100 | 3 |
| 113 | Gene_Symbol=KRT18 Keratin, type I cytoskeletal 18 | IPI00554788 | 48029 | 5,34 | 3 | 43,9 | 99 | 4 |
| 114 | Gene_Symbol=GC Vitamin D-binding protein | IPI00555812 | 54526 | 5,4 | 6 | 174,5 | 100 | 3 |
| 115 | Gene_Symbol=HPR Isoform 2 of Haptoglobin-related protein | IPI00607707 | 44054 | 6,45 | 5 | 193,9 | 100 | 4 |
| 116 | Gene_Symbol=CENPE Isoform 3 of Centromeric protein E | IPI00619925 | 302970 | 5,45 | 5 | 51,8 | 100 | 3 |
| 117 | Gene_Symbol=CREBBP CREB binding protein isoform b | IPI00619932 | 263450 | 8,78 | 3 | 55,0 | 100 | 4 |
| 118 | Gene_Symbol=KIAA1618 Isoform 1 of Protein ALO17 | IPI00642126 | 583031 | 6,05 | 6 | 60,9 | 100 | 3 |
| 119 | Gene_Symbol=MYH7B Isoform 1 of Myosin-7B | IPI00642716 | 222391 | 5,73 | 2 | 45,7 | 99 | 3 |
| 120 | Gene_Symbol=RPS4Y1 Ribosomal protein S4, Y-linked 1 | IPI00643830 | 29635 | 10,25 | 3 | 46,3 | 99 | 4 |
| 121 | Gene_Symbol=ROGDI Protein rogdi homolog | IPI00645577 | 32462 | 8,31 | 2 | 53,2 | 100 | 3 |
| 122 | Gene_Symbol=CDH3 Isoform 2 of Cadherin-3 | IPI00645614 | 87012 | 4,84 | 2 | 48,4 | 100 | 3 |
| 123 | Gene_Symbol=SAMD9L Isoform 1 of Sterile alpha motif domain-containing protein 9-like | IPI00719690 | 186126 | 8,25 | 4 | 40,2 | 97 | 4 |
| 124 | Gene_Symbol=THSD7A Thrombospondin type-1 domain-containing protein 7A | IPI00741524 | 192854 | 7,74 | 4 | 48,1 | 100 | 4 |
| 125 | Gene_Symbol=BAT2L HLA-B associated transcript 2-like | IPI00741537 | 243958 | 8,55 | 3 | 44,7 | 99 | 4 |
| 126 | Gene_Symbol=MYH9 FLJ00279 protein (Fragment) | IPI00742780 | 66015 | 8,97 | 3 | 49,7 | 100 | 4 |
| 127 | Gene_Symbol=TRAF3 TNF receptor-associated factor 3 isoform 2 | IPI00744216 | 63145 | 8,18 | 1 | 40,1 | 97 | 3 |
| 128 | Gene_Symbol=ALB Isoform 1 of Serum albumin | IPI00745872 | 71317 | 5,92 | 50 | 4357,7 | 100 | 4 |
| 129 | Gene_Symbol=BAZ2B bromodomain adjacent to zinc finger domain, 2B | IPI00747713 | 242078 | 6,13 | 3 | 44,1 | 99 | 4 |
| 130 | Gene_Symbol=C6orf163 Uncharacterized protein C6orf163 | IPI00783612 | 38757 | 6,49 | 2 | 50,9 | 100 | 4 |
| 131 | Gene_Symbol=RYR1 Isoform 1 of Ryanodine receptor 1 | IPI00783826 | 570517 | 5,18 | 5 | 80,8 | 100 | 3 |
| 132 | Gene_Symbol=JAK1 Tyrosine-protein kinase JAK1 | IPI00784013 | 135016 | 7,48 | 3 | 64,3 | 100 | 4 |
| 133 | Gene_Symbol=HSPD1 60 kDa heat shock protein, mitochondrial | IPI00784154 | 61188 | 5,7 | 2 | 53,8 | 100 | 4 |
| 134 | Gene_Symbol=DNAH10 Isoform 1 of Dynein heavy chain 10, axonemal | IPI00784869 | 517677 | 5,64 | 3 | 51,5 | 100 | 4 |
| 135 | Gene_Symbol=TBC1D23 Isoform 1 of TBC1 domain family member 23 | IPI00788879 | 79184 | 5,23 | 2 | 55,5 | 100 | 3 |
| 136 | Gene_Symbol=CCNT1 Cyclin T1b | IPI00791037 | 21307 | 7,06 | 2 | 39,8 | 97 | 4 |
| 137 | Gene_Symbol=TRPM1 Isoform 2 of Transient receptor potential cation channel subfamily M member 1 | IPI00792283 | 175504 | 6,42 | 2 | 42,2 | 98 | 3 |
| 138 | Gene_Symbol=KRT72 cDNA FLJ50908, keratin protein K6irs (K6IRS2) | IPI00793641 | 45128 | 5,11 | 3 | 98,8 | 100 | 4 |
| 139 | Gene_Symbol=COL7A1 Isoform 2 of Collagen alpha-1(VII) chain | IPI00795118 | 293060 | 5,92 | 3 | 61,4 | 100 | 4 |
| 140 | Gene_Symbol=RARG 17 kDa protein | IPI00795996 | 18031 | 10,92 | 1 | 46,8 | 99 | 4 |
| 141 | Gene_Symbol=LYST Isoform 1 of Lysosomal-trafficking regulator | IPI00796450 | 434169 | 6,15 | 6 | 57,7 | 100 | 4 |
| 142 | Gene_Symbol=KRT4 cDNA FLJ55805, highly similar to Keratin, type II cytoskeletal 4 | IPI00797452 | 52069 | 6,45 | 2 | 105,8 | 100 | 3 |
| 143 | Gene_Symbol=KIAA1370 Isoform 2 of Uncharacterized protein KIAA1370 | IPI00797998 | 109626 | 6,69 | 3 | 46,9 | 99 | 3 |
| 144 | Gene_Symbol=ACOX3 Isoform 2 of Peroxisomal acyl-coenzyme A oxidase 3 | IPI00827610 | 70329 | 8,52 | 3 | 41,0 | 98 | 4 |
| 145 | Gene_Symbol=IGHG3 Ig gamma-3 chain C region | IPI00827754 | 42287 | 8,23 | 3 | 122,1 | 100 | 4 |
| 146 | Gene_Symbol=IGL@ IGL@ protein | IPI00829626 | 25287 | 5,21 | 4 | 189,2 | 100 | 3 |
| 147 | Gene_Symbol=IGHG4 Ig gamma-4 chain C region | IPI00829814 | 36431 | 7,18 | 3 | 166,5 | 100 | 3 |
| 148 | Gene_Symbol=IGHG1 IGHG1 protein | IPI00829944 | 51906 | 7,88 | 6 | 337,4 | 100 | 4 |
| 149 | Gene_Symbol=IGKC IGKC protein | IPI00845354 | 25716 | 6,3 | 4 | 252,7 | 100 | 4 |
| 150 | Gene_Symbol=BAHCC1 BAH and coiled-coil domain-containing protein 1 | IPI00845508 | 279554 | 9,02 | 4 | 42,0 | 98 | 4 |
| 151 | Gene_Symbol=RHBDF1 cDNA FLJ60400, Rhomboid family 1 | IPI00852996 | 64499 | 10,27 | 2 | 51,3 | 100 | 4 |
| 152 | Gene_Symbol=HBA1;HBA2 Alpha 2 globin variant (Fragment) | IPI00853068 | 15328 | 8,72 | 1 | 45,4 | 99 | 4 |
| 153 | Gene_Symbol=SYDE2 synapse defective 1, Rho GTPase, homolog 2 | IPI00853256 | 134743 | 8,83 | 4 | 41,9 | 98 | 4 |
| 154 | Gene_Symbol=APOA1 Apolipoprotein A1 | IPI00853525 | 28005 | 5,8 | 2 | 115,3 | 100 | 3 |
| 155 | Gene_Symbol=MAP3K1 Mitogen-activated protein kinase kinase kinase 1 | IPI00855985 | 166419 | 7,93 | 4 | 60,2 | 100 | 4 |
| 156 | Gene_Symbol=HECTD1 HECT domain containing 1 | IPI00871372 | 292225 | 5,21 | 2 | 42,9 | 99 | 3 |
| 157 | Gene_Symbol=CCDC123 Isoform 3 of Coiled-coil domain-containing protein 123, mitochondrial | IPI00871895 | 40268 | 7,18 | 3 | 42,0 | 98 | 4 |
| 158 | Gene_Symbol=FSIP2 Isoform 1 of Fibrous sheath-interacting protein 2 | IPI00872658 | 372310 | 7,28 | 4 | 41,7 | 98 | 4 |
| 159 | Gene_Symbol=FAM75A7 Putative uncharacterized protein FAM75A7 (Fragment) | IPI00872682 | 141468 | 8,67 | 4 | 45,0 | 99 | 4 |
| 160 | Gene_Symbol=TTLL12 Tubulin tyrosine ligase-like family, member 12 | IPI00879002 | 74185 | 5,34 | 2 | 41,1 | 98 | 4 |
| 161 | Gene_Symbol=CWF19L2 Isoform 3 of CWF19-like protein 2 | IPI00879290 | 77446 | 9,37 | 3 | 40,8 | 98 | 3 |
| 162 | Gene_Symbol=LOC643677 similar to hCG2011852 | IPI00886969 | 673822 | 8,75 | 5 | 42,7 | 98 | 3 |
| 163 | Gene_Symbol=FRMPD3 similar to FERM and PDZ domain-containing protein 3 | IPI00887257 | 181782 | 8,34 | 3 | 41,8 | 98 | 4 |
| 164 | Gene_Symbol=LOC100134794 similar to keratin 8 | IPI00887509 | 12467 | 10,16 | 2 | 72,5 | 100 | 4 |
| 165 | Gene_Symbol=hCG_1774568 similar to hCG1774568 | IPI00887605 | 32051 | 11,81 | 1 | 43,3 | 99 | 4 |
| 166 | Gene_Symbol=GUCY2G Guanylyl cyclase receptor G | IPI00887656 | 123300 | 9,71 | 3 | 45,0 | 99 | 4 |
| 167 | Gene_Symbol=JAKMIP1 Isoform 5 of Janus kinase and microtubule-interacting protein 1 | IPI00887997 | 75927 | 5,03 | 3 | 49,5 | 100 | 3 |
| 168 | Gene_Symbol=LOC100129958 similar to hCG1643231 | IPI00888053 | 35448 | 8,21 | 3 | 86,6 | 100 | 4 |
| 169 | Gene_Symbol=LOC654340 similar to KIAA1839 protein | IPI00888169 | 60978 | 9,68 | 2 | 45,3 | 99 | 4 |
| 170 | Gene_Symbol=FAT4 Isoform 3 of Protocadherin Fat 4 | IPI00888207 | 546214 | 4,77 | 5 | 65,7 | 100 | 4 |
| 171 | Gene_Symbol=LOC728498 similar to golgi autoantigen, golgin subfamily a, 8A isoform 1 | IPI00888557 | 68545 | 8,92 | 4 | 63,6 | 100 | 4 |
| 172 | Gene_Symbol=LOC100129673 similar to hCG2042915 | IPI00888637 | 36144 | 11,6 | 1 | 41,5 | 98 | 3 |
| 173 | Gene_Symbol=HRNR similar to hCG1642996 | IPI00888806 | 215051 | 5,88 | 6 | 42,2 | 98 | 3 |
| 174 | Gene_Symbol=FHAD1 Isoform 1 of Forkhead-associated domain-containing protein 1 | IPI00888920 | 162659 | 6,52 | 3 | 49,2 | 100 | 3 |
| 175 | Gene_Symbol=LOC732436 similar to lipoprotein Lp | IPI00889177 | 41176 | 9,78 | 2 | 42,8 | 99 | 4 |
| 176 | Gene_Symbol=SMCHD1 Isoform 1 of Structural maintenance of chromosomes flexible hinge domain-containing protein 1 | IPI00890837 | 227942 | 6,95 | 3 | 50,6 | 100 | 3 |
| 177 | Gene_Symbol=- DMXL2 protein | IPI00896496 | 272826 | 6,12 | 2 | 40,0 | 97 | 4 |
| 178 | Gene_Symbol=HP Haptoglobin | IPI00902590 | 45861 | 6,13 | 8 | 281,6 | 100 | 4 |
| 179 | Gene_Symbol=HPR cDNA FLJ31310 fis, clone LIVER1000165 | IPI00902867 | 31673 | 8,48 | 7 | 225,4 | 100 | 4 |
| 180 | Gene_Symbol=- cDNA FLJ54284, Transcription initiation factor IIF alpha subunit | IPI00909065 | 46409 | 5,75 | 3 | 56,2 | 100 | 4 |
| 181 | Gene_Symbol=- cDNA FLJ51567, tubulin tyrosine ligase-like family, member 9 (Ttll9) | IPI00909292 | 42575 | 5,83 | 4 | 62,5 | 100 | 3 |
| 182 | Gene_Symbol=- cDNA FLJ58245, Receptor-type tyrosine-protein phosphatase epsilon | IPI00909409 | 78377 | 8,51 | 2 | 49,4 | 100 | 4 |
| 183 | Gene_Symbol=- cDNA FLJ56758, Dolichyl-phosphate beta-glucosyltransferase | IPI00909749 | 25520 | 9,14 | 4 | 63,8 | 100 | 4 |
| 184 | Gene_Symbol=KRT4 KRT4 protein (Fragment) | IPI00910053 | 11991 | 8,09 | 2 | 65,3 | 100 | 4 |
| 185 | Gene_Symbol=- cDNA FLJ58398, A-kinase anchor protein 8 | IPI00910261 | 65636 | 4,97 | 2 | 50,4 | 100 | 4 |
| 186 | Gene_Symbol=- cDNA FLJ59792, outer dense fiber of sperm tails 2-like (ODF2L), transcript variant 1 | IPI00910887 | 68103 | 6,19 | 2 | 41,4 | 98 | 4 |
| 187 | Gene_Symbol=NEB Nebulin | IPI00914847 | 775749 | 9,1 | 8 | 79,7 | 100 | 4 |
| 188 | Gene_Symbol=MAST4 Isoform 1 of Microtubule-associated serine/threonine-protein kinase 4 | IPI00914890 | 286423 | 8,85 | 5 | 72,3 | 100 | 4 |
