# Supplementary material for: An automated plasma protein fractionation design: high-throughput perspectives for proteomic analysis
Source: BMC Res Notes. 2012 Nov 1;5:612. doi: 10.1186/1756-0500-5-612 (PMC3517536; doi:10.1186/1756-0500-5-612)
Supplement: Additional file 5 — Supporting Table 3. Proteins identified in the lipophilic fraction. Protein name, accession number, pI and other additional information are reported. [file 1756-0500-5-612-S5.doc]

| **N** | **Protein Name** | **Accession Number** | **MW** | **PI** | **Peptide Count** | **Total Ion Score** | **Total Ion Score C.I. %** | **N tech replicates** |
| --- | --- | --- | --- | --- | --- | --- | --- | --- |
| 1 | Gene_Symbol=ARFGEF2 Brefeldin A-inhibited guanine nucleotide-exchange protein 2 | IPI00002186 | 204418 | 5,93 | 3 | 48,3 | 99,6 | 3 |
| 2 | Gene_Symbol=LRBA Lipopolysaccharide-responsive and beige-like anchor protein | IPI00002255 | 321639 | 5,4 | 3 | 52,1 | 99,8 | 4 |
| 3 | Gene_Symbol=ACTBL2 Beta-actin-like protein 2 | IPI00003269 | 42318 | 5,39 | 2 | 63,8 | 100,0 | 3 |
| 4 | Gene_Symbol=TRIP11 Thyroid receptor-interacting protein 11 | IPI00003515 | 228184 | 5,19 | 4 | 66,4 | 100,0 | 4 |
| 5 | Gene_Symbol=MKI67 Isoform Long of Antigen KI-67 | IPI00004233 | 360698 | 9,49 | 3 | 41,2 | 97,7 | 4 |
| 6 | Gene_Symbol=KRT24 Keratin, type I cytoskeletal 24 | IPI00004550 | 55567 | 4,89 | 3 | 88,6 | 100,0 | 4 |
| 7 | Gene_Symbol=BMS1 Ribosome biogenesis protein BMS1 homolog | IPI00006099 | 146571 | 6,04 | 1 | 41,2 | 97,7 | 3 |
| 8 | Gene_Symbol=CEP164 Isoform 1 of Centrosomal protein of 164 kDa | IPI00007293 | 164727 | 5,27 | 4 | 71,3 | 100,0 | 4 |
| 9 | Gene_Symbol=MYH13 Myosin-13 | IPI00007858 | 224681 | 5,56 | 2 | 51,9 | 99,8 | 3 |
| 10 | Gene_Symbol=KRT76 Keratin, type II cytoskeletal 2 oral | IPI00008359 | 66400 | 8,38 | 8 | 404,7 | 100,0 | 3 |
| 11 | Gene_Symbol=UTRN Utrophin | IPI00009329 | 396444 | 5,2 | 4 | 49,3 | 99,6 | 3 |
| 12 | Gene_Symbol=RANBP17 Ran-binding protein 17 | IPI00009645 | 125949 | 6,02 | 4 | 44,8 | 99,0 | 4 |
| 13 | Gene_Symbol=KRT10 Keratin, type I cytoskeletal 10 | IPI00009865 | 59703 | 5,13 | 27 | 1689,1 | 100,0 | 4 |
| 14 | Gene_Symbol=KIF5B Kinesin-1 heavy chain | IPI00012837 | 110358 | 6,12 | 4 | 44,1 | 98,9 | 3 |
| 15 | Gene_Symbol=ORC1L Origin recognition complex subunit 1 | IPI00013215 | 98372 | 9,33 | 2 | 41,8 | 98,0 | 3 |
| 16 | Gene_Symbol=DSP Isoform DPI of Desmoplakin | IPI00013933 | 334021 | 6,44 | 4 | 41,8 | 98,1 | 4 |
| 17 | Gene_Symbol=KIAA0841 Isoform 1 of Uncharacterized protein KIAA0841 | IPI00014220 | 72265 | 8,83 | 3 | 47,2 | 99,4 | 3 |
| 18 | Gene_Symbol=KRT12 Keratin, type I cytoskeletal 12 | IPI00015309 | 53592 | 4,7 | 4 | 154,5 | 100,0 | 3 |
| 19 | Gene_Symbol=C20orf39 Transmembrane protein 90B | IPI00016470 | 28761 | 4,74 | 2 | 40,1 | 97,1 | 3 |
| 20 | Gene_Symbol=MSH2 DNA mismatch repair protein Msh2 | IPI00017303 | 105418 | 5,58 | 3 | 45,3 | 99,1 | 3 |
| 21 | Gene_Symbol=KRT9 Keratin, type I cytoskeletal 9 | IPI00019359 | 62320 | 5,19 | 17 | 915,6 | 100,0 | 4 |
| 22 | Gene_Symbol=F10 Coagulation factor X | IPI00019576 | 56065 | 5,68 | 3 | 40,1 | 97,1 | 3 |
| 23 | Gene_Symbol=BSN Protein bassoon | IPI00020153 | 418354 | 7,28 | 4 | 51,6 | 99,8 | 4 |
| 24 | Gene_Symbol=SLC43A1 Isoform 1 of Large neutral amino acids transporter small subunit 3 | IPI00021075 | 62292 | 7,85 | 4 | 44,2 | 98,9 | 3 |
| 25 | Gene_Symbol=ACTA1 Actin, alpha skeletal muscle | IPI00021428 | 42366 | 5,23 | 4 | 111,5 | 100,0 | 4 |
| 26 | Gene_Symbol=ACTG1 Actin, cytoplasmic 2 | IPI00021440 | 42108 | 5,31 | 3 | 85,3 | 100,0 | 3 |
| 27 | Gene_Symbol=KIF13B Kinesin-like protein KIF13B | IPI00021753 | 203909 | 5,56 | 4 | 70,5 | 100,0 | 4 |
| 28 | Gene_Symbol=TF Serotransferrin | IPI00022463 | 79280 | 6,81 | 5 | 106,0 | 100,0 | 4 |
| 29 | Gene_Symbol=SMNDC1 Survival of motor neuron-related-splicing factor 30 | IPI00025176 | 26866 | 6,78 | 3 | 49,0 | 99,6 | 3 |
| 30 | Gene_Symbol=APC2 Isoform 1 of Adenomatous polyposis coli protein 2 | IPI00025190 | 245966 | 9,08 | 4 | 41,8 | 98,0 | 4 |
| 31 | Gene_Symbol=TNXB Isoform XB of Tenascin-X | IPI00025276 | 471178 | 5,19 | 3 | 40,0 | 97,0 | 3 |
| 32 | Gene_Symbol=MYH1 Myosin-1 | IPI00025879 | 223946 | 5,59 | 4 | 81,8 | 100,0 | 4 |
| 33 | Gene_Symbol=SF3B1 Splicing factor 3B subunit 1 | IPI00026089 | 146464 | 6,58 | 3 | 44,3 | 98,9 | 4 |
| 34 | Gene_Symbol=PTK2B Isoform 1 of Protein tyrosine kinase 2 beta | IPI00029702 | 117112 | 5,91 | 3 | 43,6 | 98,7 | 3 |
| 35 | Gene_Symbol=SPEN Msx2-interacting protein | IPI00045914 | 403030 | 7,35 | 6 | 85,7 | 100,0 | 4 |
| 36 | Gene_Symbol=CCDC102A Coiled-coil domain-containing protein 102A | IPI00059169 | 62816 | 5,44 | 2 | 46,3 | 99,3 | 3 |
| 37 | Gene_Symbol=SSH1 Isoform 3 of Protein phosphatase Slingshot homolog 1 | IPI00103741 | 105975 | 5,83 | 2 | 47,1 | 99,4 | 4 |
| 38 | Gene_Symbol=ZFYVE1 Isoform 1 of Zinc finger FYVE domain-containing protein 1 | IPI00103874 | 89458 | 7,29 | 2 | 41,8 | 98,0 | 3 |
| 39 | Gene_Symbol=PLXNB3 Plexin-B3 | IPI00155729 | 210308 | 5,96 | 2 | 41,0 | 97,6 | 3 |
| 40 | Gene_Symbol=TNRC6A Isoform 2 of Trinucleotide repeat-containing gene 6A protein | IPI00160265 | 182626 | 6,15 | 3 | 41,1 | 97,7 | 4 |
| 41 | Gene_Symbol=SDR42E1 3-beta-HSD family protein HSPC105 | IPI00163504 | 44598 | 8,43 | 2 | 49,3 | 99,7 | 4 |
| 42 | Gene_Symbol=KRT73 Isoform 1 of Keratin, type II cytoskeletal 73 | IPI00174775 | 59457 | 6,93 | 6 | 248,9 | 100,0 | 4 |
| 43 | Gene_Symbol=CWC22 Nucampholin homolog | IPI00177381 | 106041 | 6,69 | 3 | 44,5 | 98,9 | 4 |
| 44 | Gene_Symbol=TPM3 tropomyosin 3 isoform 1 | IPI00183968 | 32987 | 4,68 | 2 | 41,9 | 98,1 | 3 |
| 45 | Gene_Symbol=QTRT1 queuine tRNA-ribosyltransferase 1 | IPI00215974 | 44703 | 6,82 | 2 | 53,1 | 99,9 | 4 |
| 46 | Gene_Symbol=ASB2 Ankyrin repeat and SOCS box protein 2 | IPI00216028 | 71080 | 6,64 | 2 | 41,5 | 97,9 | 3 |
| 47 | Gene_Symbol=TPM1 Isoform 3 of Tropomyosin alpha-1 chain | IPI00216135 | 32856 | 4,72 | 3 | 61,2 | 100,0 | 4 |
| 48 | Gene_Symbol=PTK2B Isoform 2 of Protein tyrosine kinase 2 beta | IPI00216435 | 112366 | 5,69 | 4 | 40,5 | 97,1 | 4 |
| 49 | Gene_Symbol=MYL2 Myosin regulatory light chain 2, ventricular/cardiac muscle isoform | IPI00216798 | 18777 | 4,92 | 3 | 129,2 | 100,0 | 4 |
| 50 | Gene_Symbol=CCAR1 Cell division cycle and apoptosis regulator protein 1 | IPI00217357 | 133423 | 5,57 | 5 | 68,1 | 100,0 | 4 |
| 51 | Gene_Symbol=C17orf57 Isoform 1 of EF-hand domain-containing protein C17orf57 | IPI00217691 | 111143 | 6,15 | 4 | 50,4 | 99,7 | 3 |
| 52 | Gene_Symbol=TOP2B Isoform Beta-1 of DNA topoisomerase 2-beta | IPI00217709 | 183518 | 8,22 | 7 | 71,2 | 100,0 | 4 |
| 53 | Gene_Symbol=KRT16 Keratin, type I cytoskeletal 16 | IPI00217963 | 51578 | 4,99 | 14 | 555,2 | 100,0 | 4 |
| 54 | Gene_Symbol=NALCN Isoform 1 of Sodium leak channel non-selective protein | IPI00217996 | 202196 | 8,93 | 3 | 49,3 | 99,7 | 3 |
| 55 | Gene_Symbol=SPTBN2 Isoform 2 of Spectrin beta chain, brain 2 | IPI00218207 | 269709 | 5,78 | 5 | 46,1 | 99,3 | 3 |
| 56 | Gene_Symbol=ITPR1 Isoform 6 of Inositol 1,4,5-trisphosphate receptor type 1 | IPI00218662 | 315371 | 5,7 | 4 | 53,8 | 99,9 | 3 |
| 57 | Gene_Symbol=TOP2A Isoform 3 of DNA topoisomerase 2-alpha | IPI00218753 | 179398 | 8,92 | 7 | 60,2 | 100,0 | 4 |
| 58 | Gene_Symbol=ZMYM3 Isoform 2 of Zinc finger MYM-type protein 3 | IPI00219027 | 154806 | 6,01 | 4 | 48,5 | 99,6 | 4 |
| 59 | Gene_Symbol=EXOC3 Isoform 2 of Exocyst complex component 3 | IPI00219954 | 74207 | 6,71 | 2 | 44,2 | 98,9 | 3 |
| 60 | Gene_Symbol=KRT1 Keratin, type II cytoskeletal 1 | IPI00220327 | 66149 | 8,16 | 27 | 2037,7 | 100,0 | 4 |
| 61 | Gene_Symbol=AKAP9 A-kinase anchor protein 9 | IPI00220624 | 454924 | 4,94 | 4 | 41,7 | 98,0 | 4 |
| 62 | Gene_Symbol=RGS20 Isoform 1 of Regulator of G-protein signaling 20 | IPI00220880 | 32092 | 5,21 | 2 | 40,3 | 97,2 | 3 |
| 63 | Gene_Symbol=KRT79 Keratin, type II cytoskeletal 79 | IPI00241841 | 58059 | 6,75 | 6 | 370,1 | 100,0 | 4 |
| 64 | Gene_Symbol=MYL3 Myosin light chain 3 | IPI00243742 | 22089 | 5,03 | 4 | 139,3 | 100,0 | 3 |
| 65 | Gene_Symbol=DGCR6L Protein DGCR6L | IPI00260322 | 25088 | 7,03 | 1 | 40,4 | 97,3 | 3 |
| 66 | Gene_Symbol=KRT15 Keratin, type I cytoskeletal 15 | IPI00290077 | 49395 | 4,71 | 5 | 179,2 | 100,0 | 4 |
| 67 | Gene_Symbol=NPAT Protein NPAT | IPI00290547 | 155507 | 5,6 | 3 | 65,7 | 100,0 | 4 |
| 68 | Gene_Symbol=KRT3 Keratin, type II cytoskeletal 3 | IPI00290857 | 64636 | 6,12 | 7 | 382,7 | 100,0 | 4 |
| 69 | Gene_Symbol=NUMA1 Isoform 1 of Nuclear mitotic apparatus protein 1 | IPI00292771 | 239199 | 5,63 | 3 | 68,5 | 100,0 | 4 |
| 70 | Gene_Symbol=KRT83 Keratin, type II cuticular Hb3 | IPI00297795 | 55928 | 5,54 | 4 | 204,2 | 100,0 | 4 |
| 71 | Gene_Symbol=IRF7 Isoform B of Interferon regulatory factor 7 | IPI00298039 | 52225 | 6,11 | 2 | 62,4 | 100,0 | 4 |
| 72 | Gene_Symbol=PDE4C Isoform PDE4C1 of cAMP-specific 3',5'-cyclic phosphodiesterase 4C | IPI00298070 | 80422 | 5,06 | 4 | 54,6 | 99,9 | 3 |
| 73 | Gene_Symbol=KRT6C Keratin, type II cytoskeletal 6C | IPI00299145 | 60273 | 8,09 | 17 | 950,3 | 100,0 | 4 |
| 74 | Gene_Symbol=KIF14 Kinesin-like protein KIF14 | IPI00299554 | 187743 | 8,06 | 3 | 44,8 | 99,0 | 4 |
| 75 | Gene_Symbol=KRT84 Keratin, type II cuticular Hb4 | IPI00300052 | 65938 | 8 | 5 | 260,5 | 100,0 | 4 |
| 76 | Gene_Symbol=KRT82 Keratin, type II cuticular Hb2 | IPI00300053 | 57985 | 6,4 | 5 | 265,4 | 100,0 | 4 |
| 77 | Gene_Symbol=SPATA9 Isoform 2 of Spermatogenesis-associated protein 9 | IPI00300396 | 20331 | 5,96 | 2 | 44,0 | 98,8 | 3 |
| 78 | Gene_Symbol=C1orf77 Isoform 1 of Uncharacterized protein C1orf77 | IPI00300990 | 26380 | 12,24 | 3 | 49,0 | 99,6 | 3 |
| 79 | Gene_Symbol=MAPK3 mitogen-activated protein kinase 3 isoform 3 | IPI00304111 | 38536 | 5,8 | 2 | 40,7 | 97,5 | 3 |
| 80 | Gene_Symbol=CYP26A1 Cytochrome P450 26A1 | IPI00304967 | 56961 | 8,96 | 3 | 46,6 | 99,3 | 3 |
| 81 | Gene_Symbol=PRPF6 Pre-mRNA-processing factor 6 | IPI00305068 | 107656 | 8,49 | 2 | 43,9 | 98,8 | 3 |
| 82 | Gene_Symbol=NCK2 Cytoplasmic protein NCK2 | IPI00306531 | 43117 | 6,49 | 2 | 54,3 | 99,9 | 4 |
| 83 | Gene_Symbol=CHRD Isoform 1 of Chordin | IPI00306710 | 104703 | 8,07 | 1 | 41,9 | 98,1 | 4 |
| 84 | Gene_Symbol=ZNF518A Isoform 1 of Zinc finger protein 518A | IPI00307665 | 169071 | 9,38 | 3 | 45,0 | 99,1 | 3 |
| 85 | Gene_Symbol=GIPC3 PDZ domain-containing protein GIPC3 | IPI00307688 | 34302 | 5,5 | 2 | 40,0 | 97,0 | 3 |
| 86 | Gene_Symbol=KRT27 Keratin, type I cytoskeletal 27 | IPI00328103 | 50420 | 5,06 | 4 | 198,7 | 100,0 | 4 |
| 87 | Gene_Symbol=OTOP3 Otopetrin-3 | IPI00332628 | 67222 | 8,96 | 1 | 45,2 | 99,1 | 4 |
| 88 | Gene_Symbol=GCC2 Isoform 2 of GRIP and coiled-coil domain-containing protein 2 | IPI00333197 | 196872 | 5,1 | 2 | 40,3 | 97,2 | 3 |
| 89 | Gene_Symbol=HMMR hyaluronan-mediated motility receptor isoform a | IPI00337772 | 84576 | 5,73 | 2 | 41,5 | 97,9 | 4 |
| 90 | Gene_Symbol=KRT26 Keratin, type I cytoskeletal 26 | IPI00375910 | 52620 | 4,86 | 3 | 83,9 | 100,0 | 4 |
| 91 | Gene_Symbol=PRKDC Isoform 2 of DNA-dependent protein kinase catalytic subunit | IPI00376215 | 470162 | 6,81 | 3 | 78,6 | 100,0 | 4 |
| 92 | Gene_Symbol=KRT77 keratin 77 | IPI00376379 | 62149 | 5,73 | 4 | 312,9 | 100,0 | 4 |
| 93 | Gene_Symbol=GFAP Isoform 2 of Glial fibrillary acidic protein | IPI00383815 | 50372 | 5,53 | 4 | 155,1 | 100,0 | 4 |
| 94 | Gene_Symbol=KRT14 Keratin, type I cytoskeletal 14 | IPI00384444 | 51875 | 5,09 | 14 | 468,0 | 100,0 | 4 |
| 95 | Gene_Symbol=CCDC102B Isoform 3 of Coiled-coil domain-containing protein 102B | IPI00385048 | 49683 | 5,86 | 2 | 41,3 | 97,8 | 3 |
| 96 | Gene_Symbol=SYNE1 Isoform 1 of Nesprin-1 | IPI00386444 | 1017083 | 5,38 | 8 | 75,6 | 100,0 | 3 |
| 97 | Gene_Symbol=ACLY cDNA FLJ56442, ATP-citrate synthase | IPI00394838 | 125970 | 8,4 | 3 | 47,9 | 99,5 | 3 |
| 98 | Gene_Symbol=GRM8 Isoform B of Metabotropic glutamate receptor 8 | IPI00396012 | 103140 | 8,49 | 1 | 40,4 | 97,3 | 4 |
| 99 | Gene_Symbol=ZNF844 Zinc finger protein 844 | IPI00397691 | 78708 | 9,14 | 2 | 61,3 | 100,0 | 4 |
| 100 | Gene_Symbol=RNASE2 zinc finger protein 749 | IPI00397740 | 92701 | 9,01 | 2 | 42,1 | 98,2 | 4 |
| 101 | Gene_Symbol=PLEC1 Isoform 3 of Plectin-1 | IPI00398002 | 519655 | 5,59 | 8 | 61,7 | 100,0 | 4 |
| 102 | Gene_Symbol=LUC7L Isoform 2 of Putative RNA-binding protein Luc7-like 1 | IPI00410026 | 38781 | 10,06 | 2 | 45,6 | 99,2 | 3 |
| 103 | Gene_Symbol=ZDHHC17 Isoform 3 of Palmitoyltransferase ZDHHC17 | IPI00410688 | 29933 | 5,66 | 1 | 43,3 | 98,6 | 4 |
| 104 | Gene_Symbol=KIF20B Isoform 3 of M-phase phosphoprotein 1 | IPI00412862 | 207373 | 5,64 | 4 | 52,7 | 99,8 | 3 |
| 105 | Gene_Symbol=MYL6B 11 kDa protein | IPI00413922 | 10825 | 4,84 | 1 | 41,8 | 98,0 | 3 |
| 106 | Gene_Symbol=- HERV-K_12q14.1 provirus ancestral Env polyprotein | IPI00414889 | 80154 | 9,22 | 2 | 43,8 | 98,8 | 3 |
| 107 | Gene_Symbol=CCDC73 Isoform 1 of Coiled-coil domain-containing protein 73 | IPI00418774 | 125046 | 5,42 | 3 | 47,9 | 99,5 | 3 |
| 108 | Gene_Symbol=SHROOM3 PDZ domain actin binding protein Shroom mRNA | IPI00446280 | 88375 | 9,56 | 2 | 41,0 | 97,7 | 3 |
| 109 | Gene_Symbol=DYNC1H1 Cytoplasmic dynein 1 heavy chain 1 | IPI00456969 | 534809 | 6,01 | 6 | 56,5 | 99,9 | 4 |
| 110 | Gene_Symbol=VPS13C Isoform 1 of Vacuolar protein sorting-associated protein 13C | IPI00465428 | 424461 | 6,38 | 3 | 71,1 | 100,0 | 4 |
| 111 | Gene_Symbol=PLXND1 Isoform 2 of Plexin-D1 | IPI00472139 | 198828 | 7 | 4 | 46,6 | 99,4 | 3 |
| 112 | Gene_Symbol=- 88 kDa protein | IPI00472777 | 89480 | 7,77 | 2 | 45,6 | 99,2 | 3 |
| 113 | Gene_Symbol=DNAH8 Isoform 2 of Dynein heavy chain 8, axonemal | IPI00478021 | 513657 | 5,92 | 3 | 49,2 | 99,6 | 3 |
| 114 | Gene_Symbol=STIP1 STIP1 protein | IPI00479946 | 68687 | 7,81 | 3 | 46,4 | 99,3 | 4 |
| 115 | Gene_Symbol=MYH6 Myosin-6 | IPI00514201 | 224393 | 5,6 | 12 | 290,7 | 100,0 | 4 |
| 116 | Gene_Symbol=MACF1 670 kDa protein | IPI00514468 | 673696 | 5,2 | 3 | 41,9 | 98,1 | 3 |
| 117 | Gene_Symbol=DAAM2 Disheveled-associated activator of morphogenesis 2 | IPI00514893 | 124276 | 6,36 | 3 | 40,7 | 97,5 | 4 |
| 118 | Gene_Symbol=CEP135 Isoform 1 of Centrosomal protein of 135 kDa | IPI00550987 | 133878 | 5,87 | 2 | 44,5 | 99,0 | 4 |
| 119 | Gene_Symbol=MLLT4 Isoform 1 of Afadin | IPI00552765 | 182855 | 6,1 | 4 | 48,0 | 99,5 | 4 |
| 120 | Gene_Symbol=THOC2 THO complex 2 | IPI00552909 | 44212 | 9,64 | 3 | 46,0 | 99,2 | 3 |
| 121 | Gene_Symbol=REV1 Isoform 2 of DNA repair protein REV1 | IPI00552928 | 139288 | 8,76 | 3 | 45,1 | 99,1 | 3 |
| 122 | Gene_Symbol=ATR Isoform 2 of Serine/threonine-protein kinase ATR | IPI00554573 | 297451 | 7,16 | 3 | 47,7 | 99,5 | 4 |
| 123 | Gene_Symbol=KRT8 Keratin, type II cytoskeletal 8 | IPI00554648 | 53671 | 5,52 | 5 | 355,4 | 100,0 | 4 |
| 124 | Gene_Symbol=KRT18 Keratin, type I cytoskeletal 18 | IPI00554788 | 48029 | 5,34 | 3 | 58,5 | 100,0 | 4 |
| 125 | Gene_Symbol=EXO1 Isoform 2 of Exonuclease 1 | IPI00556476 | 90201 | 8,69 | 3 | 40,5 | 97,3 | 4 |
| 126 | Gene_Symbol=NARG2 Isoform 2 of NMDA receptor-regulated protein 2 | IPI00604683 | 95516 | 5,91 | 2 | 43,1 | 98,5 | 3 |
| 127 | Gene_Symbol=GRIPAP1 Isoform 4 of GRIP1-associated protein 1 | IPI00607628 | 93023 | 5,14 | 2 | 45,1 | 99,1 | 3 |
| 128 | Gene_Symbol=MYH14 Isoform 4 of Myosin-14 | IPI00607778 | 204027 | 5,99 | 1 | 40,5 | 97,4 | 3 |
| 129 | Gene_Symbol=MYCBP2 Isoform 2 of Probable E3 ubiquitin-protein ligase MYCBP2 | IPI00607852 | 517480 | 6,63 | 5 | 45,3 | 99,1 | 3 |
| 130 | Gene_Symbol=GLG1 Isoform 1 of Golgi apparatus protein 1 | IPI00641153 | 138341 | 6,52 | 2 | 51,3 | 99,8 | 3 |
| 131 | Gene_Symbol=COLEC12 67 kDa protein | IPI00641439 | 67278 | 6,39 | 3 | 49,5 | 99,7 | 3 |
| 132 | Gene_Symbol=IL17RD Isoform 1 of Interleukin-17 receptor D | IPI00641705 | 83726 | 6,78 | 3 | 42,4 | 98,3 | 3 |
| 133 | Gene_Symbol=ZMYM4 Isoform 4 of Zinc finger MYM-type protein 4 | IPI00642195 | 172549 | 6,59 | 3 | 45,4 | 99,1 | 4 |
| 134 | Gene_Symbol=DST Dystonin | IPI00642259 | 862070 | 5,13 | 4 | 50,3 | 99,7 | 3 |
| 135 | Gene_Symbol=PDPR cDNA FLJ36072 fis, clone TESTI2019669, Hyruvate dehydrogenase phosphatase regulatory subunit | IPI00643433 | 25975 | 6,08 | 3 | 49,0 | 99,6 | 3 |
| 136 | Gene_Symbol=CDH3 Isoform 2 of Cadherin-3 | IPI00645614 | 87012 | 4,84 | 3 | 63,1 | 100,0 | 4 |
| 137 | Gene_Symbol=HYDIN2;HYDIN Isoform 1 of Hydrocephalus-inducing protein homolog | IPI00647188 | 580927 | 5,74 | 3 | 44,4 | 98,9 | 4 |
| 138 | Gene_Symbol=CHD8 Isoform 2 of Chromodomain-helicase-DNA-binding protein 8 | IPI00719073 | 277927 | 6,49 | 2 | 44,6 | 99,0 | 3 |
| 139 | Gene_Symbol=PPFIA2 PPFIA2 protein | IPI00719091 | 141638 | 5,83 | 3 | 42,5 | 98,3 | 3 |
| 140 | Gene_Symbol=WHDC1L2 WAS protein homology region 2 domain containing 1-like 2 | IPI00737049 | 71129 | 9,14 | 4 | 54,6 | 99,9 | 4 |
| 141 | Gene_Symbol=ASPM Isoform 1 of Abnormal spindle-like microcephaly-associated protein | IPI00743813 | 413189 | 10,45 | 5 | 43,2 | 98,6 | 3 |
| 142 | Gene_Symbol=CUL9 Isoform 1 of p53-associated parkin-like cytoplasmic protein | IPI00744001 | 285440 | 5,3 | 2 | 48,6 | 99,6 | 3 |
| 143 | Gene_Symbol=ALB Isoform 1 of Serum albumin | IPI00745872 | 71317 | 5,92 | 40 | 2290,1 | 100,0 | 4 |
| 144 | Gene_Symbol=RAI14 Isoform 1 of Ankycorbin | IPI00759532 | 110601 | 5,87 | 3 | 55,2 | 99,9 | 4 |
| 145 | Gene_Symbol=WDR22 Isoform 1 of WD repeat-containing protein 22 | IPI00783277 | 104982 | 5,48 | 3 | 51,6 | 99,8 | 4 |
| 146 | Gene_Symbol=CHL1 Isoform 1 of Neural cell adhesion molecule L1-like protein | IPI00783390 | 136026 | 5,51 | 3 | 44,6 | 99,0 | 3 |
| 147 | Gene_Symbol=KIAA1324L Isoform 1 of UPF0577 protein KIAA1324-like | IPI00784017 | 117132 | 5,76 | 2 | 47,4 | 99,5 | 3 |
| 148 | Gene_Symbol=HSPD1 60 kDa heat shock protein, mitochondrial | IPI00784154 | 61188 | 5,7 | 2 | 58,5 | 100,0 | 3 |
| 149 | Gene_Symbol=CEP290 Isoform 1 of Centrosomal protein of 290 kDa | IPI00784201 | 291050 | 5,75 | 6 | 51,3 | 99,8 | 4 |
| 150 | Gene_Symbol=CSAD cysteine sulfinic acid decarboxylase | IPI00789682 | 58716 | 6,17 | 3 | 42,2 | 98,2 | 3 |
| 151 | Gene_Symbol=KRT13 31 kDa protein | IPI00791156 | 30866 | 4,99 | 8 | 263,9 | 100,0 | 4 |
| 152 | Gene_Symbol=KRT13 42 kDa protein | IPI00791852 | 41729 | 6,3 | 8 | 225,8 | 100,0 | 4 |
| 153 | Gene_Symbol=- 14 kDa protein | IPI00793191 | 14545 | 5,65 | 5 | 131,8 | 100,0 | 4 |
| 154 | Gene_Symbol=KRT72 cDNA FLJ50908, keratin protein K6irs (K6IRS2), mRNA | IPI00793641 | 45128 | 5,11 | 4 | 198,0 | 100,0 | 4 |
| 155 | Gene_Symbol=RB1CC1 Rb1-inducible coiled coil protein 1 isoform 2 | IPI00794069 | 184841 | 5,31 | 3 | 41,8 | 98,1 | 3 |
| 156 | Gene_Symbol=- 227 kDa protein | IPI00794080 | 227775 | 5,63 | 4 | 73,2 | 100,0 | 4 |
| 157 | Gene_Symbol=ZNF621 17 kDa protein | IPI00795238 | 17429 | 6,43 | 3 | 47,3 | 99,4 | 3 |
| 158 | Gene_Symbol=RPL4 60S ribosomal protein L4 | IPI00795303 | 46269 | 11,36 | 4 | 45,9 | 99,2 | 4 |
| 159 | Gene_Symbol=KRT31 51 kDa protein | IPI00796364 | 52118 | 4,97 | 3 | 203,6 | 100,0 | 4 |
| 160 | Gene_Symbol=KRT5 cDNA FLJ54081, Keratin, type II cytoskeletal 5 | IPI00796776 | 60317 | 5,95 | 11 | 584,4 | 100,0 | 4 |
| 161 | Gene_Symbol=KRT4 cDNA FLJ55805, Keratin, type II cytoskeletal 4 | IPI00797452 | 52069 | 6,45 | 3 | 193,4 | 100,0 | 4 |
| 162 | Gene_Symbol=MYBPC3 Myosin-binding protein C, cardiac-type | IPI00798035 | 141715 | 6,24 | 4 | 49,3 | 99,7 | 3 |
| 163 | Gene_Symbol=COBL Isoform 2 of Protein cordon-bleu | IPI00807589 | 144752 | 7,03 | 2 | 51,5 | 99,8 | 3 |
| 164 | Gene_Symbol=MICALCL Isoform 3 of MICAL C-terminal-like protein | IPI00844270 | 73692 | 8,76 | 4 | 63,8 | 100,0 | 3 |
| 165 | Gene_Symbol=OVGP1 Oviduct-specific glycoprotein variant (Fragment) | IPI00844591 | 82932 | 8,42 | 3 | 40,1 | 97,1 | 3 |
| 166 | Gene_Symbol=C9orf117 Isoform 2 of Uncharacterized protein C9orf117 | IPI00847343 | 50122 | 9,16 | 3 | 46,0 | 99,2 | 4 |
| 167 | Gene_Symbol=LBA1 Lupus brain antigen 1 homolog | IPI00847543 | 340341 | 6,34 | 3 | 55,9 | 99,9 | 4 |
| 168 | Gene_Symbol=CCDC138 Isoform 2 of Coiled-coil domain-containing protein 138 | IPI00847886 | 66875 | 8,67 | 2 | 40,6 | 97,4 | 3 |
| 169 | Gene_Symbol=ADAD1 Isoform 2 of Adenosine deaminase domain-containing protein 1 | IPI00852654 | 63378 | 9,06 | 2 | 43,6 | 98,7 | 3 |
| 170 | Gene_Symbol=RHBDF1 rhomboid family 1 (Drosophila) (RHBDF1), mRNA | IPI00852996 | 64499 | 10,27 | 2 | 58,0 | 100,0 | 4 |
| 171 | Gene_Symbol=TMEM131 RW1 protein | IPI00853149 | 205809 | 8,74 | 4 | 51,6 | 99,8 | 3 |
| 172 | Gene_Symbol=LMOD1 Isoform 1 of Leiomodin-1 | IPI00872593 | 67173 | 9,33 | 4 | 41,6 | 97,9 | 3 |
| 173 | Gene_Symbol=KANK4 107 kDa protein | IPI00872608 | 107886 | 5,13 | 2 | 41,2 | 97,7 | 3 |
| 174 | Gene_Symbol=KRT13 46 kDa protein | IPI00873598 | 46085 | 4,76 | 4 | 154,5 | 100,0 | 4 |
| 175 | Gene_Symbol=MYH11 Myosin heavy chain 11 smooth muscle isoform | IPI00873982 | 235003 | 5,64 | 5 | 99,1 | 100,0 | 4 |
| 176 | Gene_Symbol=CELSR1 Putative uncharacterized protein CELSR1 | IPI00874160 | 334298 | 5,58 | 3 | 64,4 | 100,0 | 4 |
| 177 | Gene_Symbol=CCDC11 48 kDa protein | IPI00874191 | 48681 | 8,61 | 3 | 43,2 | 98,6 | 3 |
| 178 | Gene_Symbol=KIF7 95 kDa protein | IPI00877116 | 95411 | 8 | 3 | 50,7 | 99,7 | 3 |
| 179 | Gene_Symbol=DNAH1 Isoform 1 of Dynein heavy chain 1, axonemal | IPI00878816 | 497704 | 5,66 | 3 | 52,9 | 99,8 | 4 |
| 180 | Gene_Symbol=- 40 kDa protein | IPI00879183 | 40450 | 9,87 | 2 | 55,4 | 99,9 | 3 |
| 181 | Gene_Symbol=- 29 kDa protein | IPI00879936 | 28920 | 4,83 | 3 | 164,0 | 100,0 | 4 |
| 182 | Gene_Symbol=CCDC88B Isoform 4 of Coiled-coil domain-containing protein 88B | IPI00884966 | 151485 | 5 | 2 | 41,8 | 98,0 | 3 |
| 183 | Gene_Symbol=DNHL1, Isoform 1 of Dynein heavy chain 6, axonemal | IPI00884996 | 479671 | 5,72 | 4 | 41,5 | 97,9 | 3 |
| 184 | Gene_Symbol=STARD9 StAR-related lipid transfer (START) domain containing 9 | IPI00886783 | 477544 | 5,8 | 5 | 95,4 | 100,0 | 4 |
| 185 | Gene_Symbol=LOC100129346 similar to FLJ42875 protein | IPI00886836 | 16885 | 11,59 | 3 | 41,9 | 98,1 | 3 |
| 186 | Gene_Symbol=LOC731605 hypothetical LOC731605 | IPI00886987 | 100703 | 11,24 | 3 | 57,2 | 99,9 | 3 |
| 187 | Gene_Symbol=LOC100134794 similar to keratin 8 | IPI00887509 | 12467 | 10,16 | 4 | 186,2 | 100,0 | 4 |
| 188 | Gene_Symbol=LOC100129958 similar to hCG1643231 | IPI00888053 | 35448 | 8,21 | 2 | 119,4 | 100,0 | 4 |
| 189 | Gene_Symbol=LOC728498 similar to golgi autoantigen, golgin subfamily a, 8A isoform 1 | IPI00888557 | 68545 | 8,92 | 2 | 45,3 | 99,1 | 4 |
| 190 | Gene_Symbol=LOC100130533 similar to hCG2012269 | IPI00888845 | 91624 | 8,43 | 3 | 41,7 | 98,0 | 3 |
| 191 | Gene_Symbol=KIF15 Isoform 2 of Kinesin-like protein KIF15 | IPI00889513 | 150489 | 5,78 | 5 | 74,4 | 100,0 | 3 |
| 192 | Gene_Symbol=- DMXL2 protein | IPI00896496 | 272828 | 6,12 | 2 | 43,4 | 98,6 | 4 |
| 193 | Gene_Symbol=- cDNA FLJ43948 fis, cytoplasmic FMR1 interacting protein 1 (CYFIP1), transcript variant 1 | IPI00903062 | 147082 | 7,54 | 3 | 46,0 | 99,2 | 3 |
| 194 | Gene_Symbol=- cDNA FLJ58398, A-kinase anchor protein 8 | IPI00910261 | 65636 | 4,97 | 2 | 44,1 | 98,9 | 4 |
| 195 | Gene_Symbol=NEFH Neurofilament, heavy polypeptide 200kDa | IPI00910602 | 112639 | 5,99 | 3 | 66,4 | 100,0 | 4 |
| 196 | Gene_Symbol=- cDNA FLJ59939, Protein disulfide-isomerase | IPI00911004 | 25146 | 9,68 | 2 | 43,8 | 98,8 | 3 |
| 197 | Gene_Symbol=- cDNA FLJ58331, glycerophosphodiester phosphodiesterase domain containing 2 (GDPD2) | IPI00911110 | 67860 | 8,81 | 2 | 40,2 | 97,2 | 3 |
| 198 | Gene_Symbol=ANKRD11 Ankyrin repeat domain-containing protein 11 | IPI00914930 | 299684 | 6,7 | 5 | 61,4 | 100,0 | 4 |
